# Complex motion of Greenland Ice Sheet outlet glaciers with basal temperate ice

**DOI:** 10.1126/sciadv.abq5180

**Published:** 2023-02-10

**Authors:** Robert Law, Poul Christoffersen, Emma MacKie, Samuel Cook, Marianne Haseloff, Olivier Gagliardini

**Affiliations:** ^1^ Scott Polar Research Institute, University of Cambridge, Cambridge, UK.; ^2^ Department of Geological Sciences, University of Florida, Gainesville, FL, USA.; ^3^ Institute of Earth Surface Dynamics, Université de Lausanne, Lausanne, Switzerland.; ^4^ Department of Geoscience, University of Wisconsin-Madison, Madison, WI, USA.; ^5^ CNRS, IRD, Grenoble INP, IGE, Université Grenoble Alpes, Grenoble, France.

## Abstract

Uncertainty associated with ice sheet motion plagues sea level rise predictions. Much of this uncertainty arises from imperfect representations of physical processes including basal slip and internal ice deformation, with ice sheet models largely incapable of reproducing borehole-based observations. Here, we model isolated three-dimensional domains from fast-moving (Sermeq Kujalleq/Store Glacier) and slow-moving (Isunnguata Sermia) ice sheet settings in Greenland. By incorporating realistic geostatistically simulated topography, we show that a spatially highly variable layer of temperate ice (much softer ice at the pressure-melting point) forms naturally in both settings, alongside ice motion patterns which diverge substantially from those obtained using smoothly varying BedMachine topography. Temperate ice is vertically extensive (>100 meters) in deep troughs but thins notably (<5 meters) over bedrock highs, with basal slip rates reaching >90 or <5% of surface velocity dependent on topography and temperate layer thickness. Developing parameterizations of the net effect of this complex motion can improve the realism of predictive ice sheet models.

## Teaser

Geostatistically realistic topography forces complex patterns of basal slip and deformation in Greenland outlet glaciers.

## INTRODUCTION

The Greenland Ice Sheet (GrIS) has transitioned from a state of near zero mass loss in the 1990s to large and sustained (>200 Gt a^−1^) annual mass losses since the mid-2000s and is now the largest cryospheric contributor to sea level rise (*1*). While the satellite era has greatly increased the accuracy of mass-balance observations, model predictions for future ice loss remain highly uncertain (*2*, *3*) but indicate substantial and nonlinear sea level rise under future anthropogenic warming (*4*–*6*). Ice sheet dynamics and their parameterization for large ice sheet models (*7*–*10*) are crucial components of this uncertainty, being responsible for ice transport to lower and warmer elevations where surface melt rates and runoff increase rapidly and to the fronts of marine-terminating glaciers where ~50% of GrIS net annual mass loss occurs through calving (*11*).

**Fig. 1.**
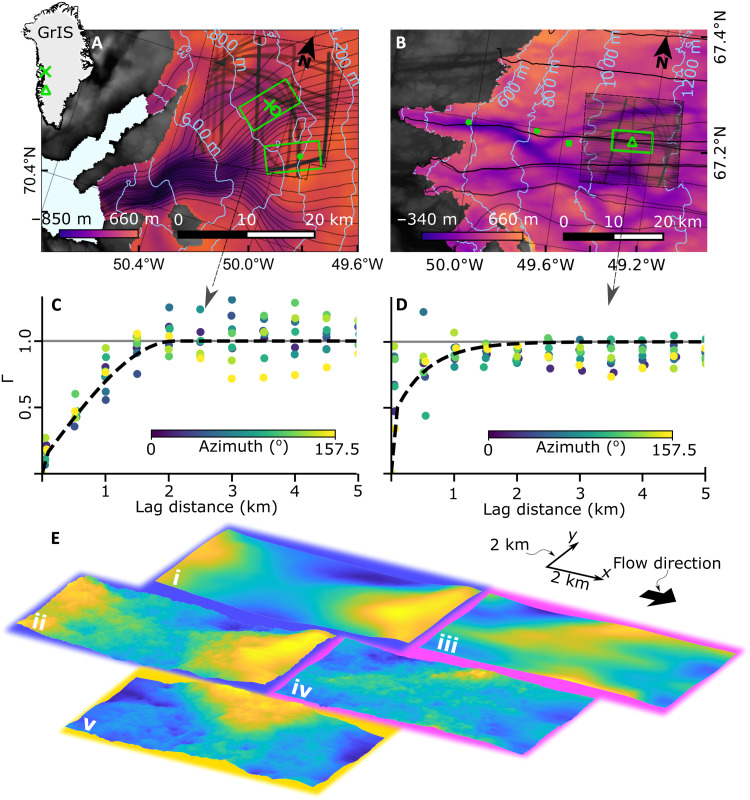
Location of modeling domains, variograms, and model setup. (**A**) Sermeq Kujalleq (Store Glacier) showing flowlines in black converging into Uummannaq Fjord. BedMachine v3 (*12*) basal topography (inferno colormap), land topography (grayscale), and ice surface contours (pale blue). Model domain locations containing RESPONDER (north fluorescent green rectangle), borehole BH19c location (fluorescent green cross) (*19*), borehole BH18c location (fluorescent circle) (*64*), SAFIRE domain (south fluorescent green rectangle), borehole BH14b-c location (fluorescent green dot) (*25*), and radar flight lines for RESPONDER domain (bold black strokes within dashed boundary, scatter opacity means darker lines have more measurements) (*61*). (**B**) As for (A) but Isunnguata Sermia showing the S5 domain (fluorescent green rectangle) and boreholes S5 (fluorescent green triangle), S4 (west fluorescent green dot), S2 (east fluorescent green dot), and IS2015 (fluorescent green square) (*20*). S2 to S5 are from (*26*). (**C**) Modeled variogram (dashed line) and empirical variograms for varying azimuths (points) for RESPONDER domain (see fig. S1 for SAFIRE variogram and flight lines). Variograms describe the spatial statistics of measured topography. (**D**) As for (C) but for Isunnguata Sermia domain. (**E**) BedMachine (i and iii) and geostatistically simulated (ii, iv, and v) basal DEMs with periodic taper applied for RESPONDER (blue outline), Isunnguata Sermia (pink outline), and SAFIRE (yellow outline) domains. Flow direction and *x*-*y* scale in top right. No vertical exaggeration used. Elevation ranges for (i) to (v) are 369, 524, 163, 320, and 755 m, respectively.

**Fig. 2.**
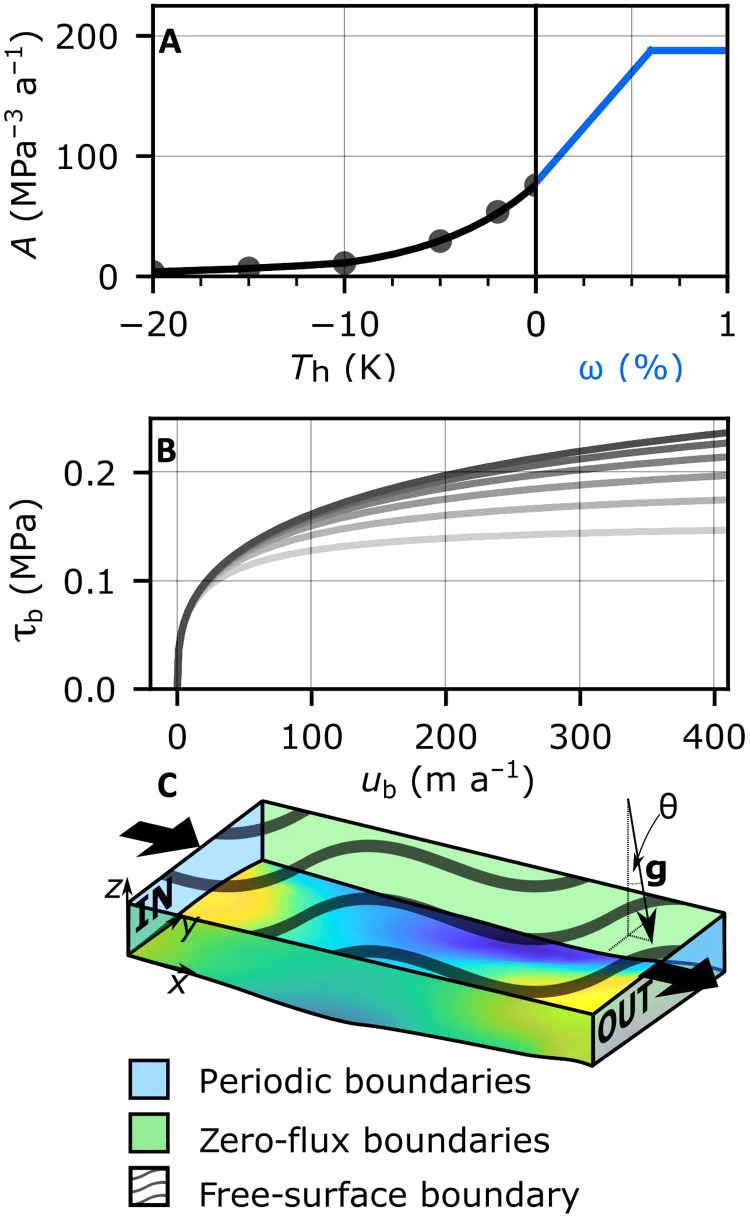
Ice rheology, basal traction, and periodic setup. (**A**) Rate parameter, *A*, as a function of homologous temperature (temperature below the melting point; black line) and water content (blue line). Black dots show values from (*65*). (**B**) Regularized Coulomb relationship with *F*= 1.2, *s* − *b*= 1043 m, *C*= 0.1617, and θ= 0.8 to 1.8° in 0.2° increments (see Materials and Methods for equation and symbol definition). (**C**) Model setup showing inflow and outflow boundaries (labeled IN and OUT), which are periodic for initial model runs [free-surface runs (FS runs) described in Materials and Methods] with RESPONDER BedMachine topography (MATLAB parula colormap), axis orientation, zero-flux lateral boundaries, free surface, and gravity vector.

Uncertainty related to ice sheet motion arises from inadequate understanding of its two major components: (i) basal slip at the ice-sediment or ice-rock interface and (ii) deformation within the ice sheet itself. State-of-the-art GrIS models run with BedMachine, the most advanced gridded data product of GrIS basal topography, which is relatively smooth compared to deglaciated terrain (*12*, *13*), produce basal slip and ice deformation rates that vary smoothly and are largely independent of one another [e.g., (*4*, *14*)]. However, GrIS borehole records indicate substantial variation in ice deformation, particularly toward the ice sheet bed (*15*–*17*) and notable catchment-scale variations in the thickness of a much softer and relatively poorly understood, basal temperate layer in which ice coexists with a liquid water phase at the pressure-dependent melting point (*18*, *19*). Here, we advance upon two-dimensional (2D) models that begin to unpick this complexity (*20*, *21*) by incorporating realistic 3D geostatistically simulated bed topography (Fig. 1) and improved temperate ice rheology in a 3D full-Stokes model (Fig. 2A). We focus on ice motion at the previously overlooked intermediate scale (≥25 m, ≲4 km), bridging recent advances in understanding at small (*22*, *23*) and large (*24*) scales. The outcomes explain why field observations can be highly variable over even short distances. The behavior we model is characterized by spatially complex patterns of modeled ice deformation—focused toward the ice sheet bed—and basal slip. The basal temperate layer is an important modulator of ice motion, extending or compressing in response to topographic perturbations, with vertical gradients in ice velocity notably reduced just above the cold-temperate transition surface (CTS). Considered over larger scales (~1 km), the behavior we report diverges substantially from the ice motion patterns presently implicitly incorporated into grid or mesh cells in state-of-the-art ice sheet models. We suggest that reconciling this improved understanding through more accurate parameterizations of ice sheet motion at large scales may lead to more accurate predictions of sea level rise in the coming decades and centuries.

## RESULTS

Our modeling approach explores ice motion in isolated domains across three distinct glaciological settings. Two domains are from the fast-moving (~500 m a^−1^) Sermeq Kujalleq (or Store Glacier; Fig. 1A), which flows into Uummannaq Fjord in West Greenland. The RESPONDER simulation is centered on the 1043-m-deep RESPONDER project borehole BH19c (*19*) drilled at the center of a drained lake above a basal topographic saddle (Fig. 1E, i and ii). The SAFIRE simulation is centered on the 611-m-deep SAFIRE project borehole BH16c (*25*), which measures ice motion over a contrasting ~300-m bedrock rise (Fig. 1E, v). The third simulation, S5, is centered over the ~818-m S5 borehole site from (*26*) on the slow-moving (<125 m a^−1^) land-terminating Isunnguata Sermia [Fig. 1, B and E (iii and iv)] where there are no substantial large-scale topographic troughs or rises.

All domains are run using geostatistically realistic topography (produced at a horizontal resolution of 20 m) using the sequential Gaussian simulation method. This well-established procedure (*27*, *28*) treats topography as a Gaussian process, thereby matching airborne radar measurements of bed elevation along flight lines exactly while also reproducing the roughness characteristics observed along flight lines (see Materials and Methods). The RESPONDER and S5 domains are additionally run using 400-m horizontal resolution BedMachine v3 topography to assess the difference in ice motion behavior resulting from the two topographic approaches. In the areas around our domains, BedMachine is derived from interpolated radar flight lines taking into account mass conservation (*29*). While this methodology is a substantial improvement over earlier kriging interpolation, the resulting topography product is still considerably smoother than topography observed along radar flight lines (*30*). We achieve a close fit between modeled and observed surface velocity in a two-step approach. First, the ice temperature and rheology throughout the domain are set from a prescribed vertical temperature profile based on a borehole record taken from the center of the domain, with minor adjustments to just remove the temperate ice layer. The effective slope of the bed topography is then gradually increased to match the observed surface velocity (Fig. 2C and table S1). Basal slip is calculated using a regularized Coulomb relationship that parameterizes complex small-scale (<25 m) behavior such as cavitation (*23*) and sediment ploughing, with fixed friction parameters for each model run [Fig. 2C, table S1, and Materials and Methods (*22*)]. This approach avoids a basal-traction inversion procedure, which masks basal variation at sub-ice thickness scales. Subsequently, we incorporate thermomechanical coupling, where the enthalpy field and, hence, ice rheology are coupled to ice advection, conduction, and strain heating. The surface and inflow boundary conditions are fixed for this stage, with the inflow temperature again set using the same borehole record from the center of the domain (see Materials and Methods).

### Ice motion through a topographic saddle (RESPONDER domain)

**Fig. 3.**
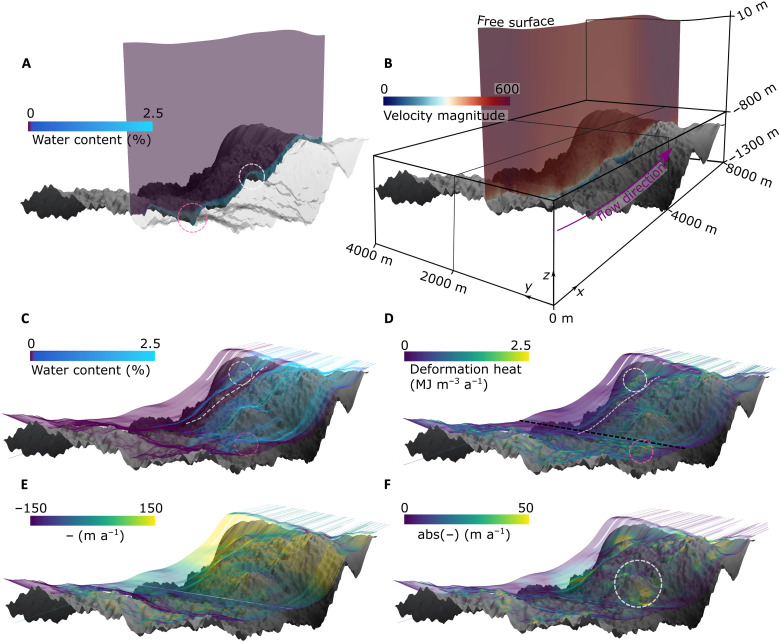
3D model output from RESPONDER geostatistical simulation (Rgb). Flow direction is left to right along the *x* axis, basal topography is in gray (maximum and minimum elevations are −835 and −1349 m, respectively). *z* axis is exaggerated by a factor of 3. (**A**) Water content and temperate ice thickness along *xz* transect intersecting *y* coordinate 1300 m (same plane as Fig. 4). Transparency applied to topography on the observer’s side of the transect. Pink dashed ring in (A) highlights area of thickened temperate ice in topographic trough, while white dashed ring in (A) highlights area of thinned temperate ice over topographic rise. Purple here and in (C) indicates water content is 0. (**B**) Transect as for (A) showing velocity magnitude with flow direction in pink, axis orientation and dimensions visible. (**C**) Water content mapped onto 750 flowlines originating at line with coordinates [(3000, 0, −1083.3), (3000, 4000, −1083.3)] shown as black dashed line in (D). (**D**) As for (C) but with deformation heat. Pink dashed rings in (C) and (D) highlight high but variable deformation heating where particles are close to the base over rough topography. White dashed rings in (C) and (D) highlight high deformation heating over a topographic prominence. White dashed lines in (C) and (D) highlight an area of cold ice with low deformation heating. (**E**) As for (C) but *z* component of velocity vector mapped onto flowlines. (**F**) As for (C) but magnitude of *y* component of velocity vector mapped onto flowlines. White ring in (F) highlights region of high abs(*u_y_*) around an area of high topographic prominence.

**Fig. 4.**
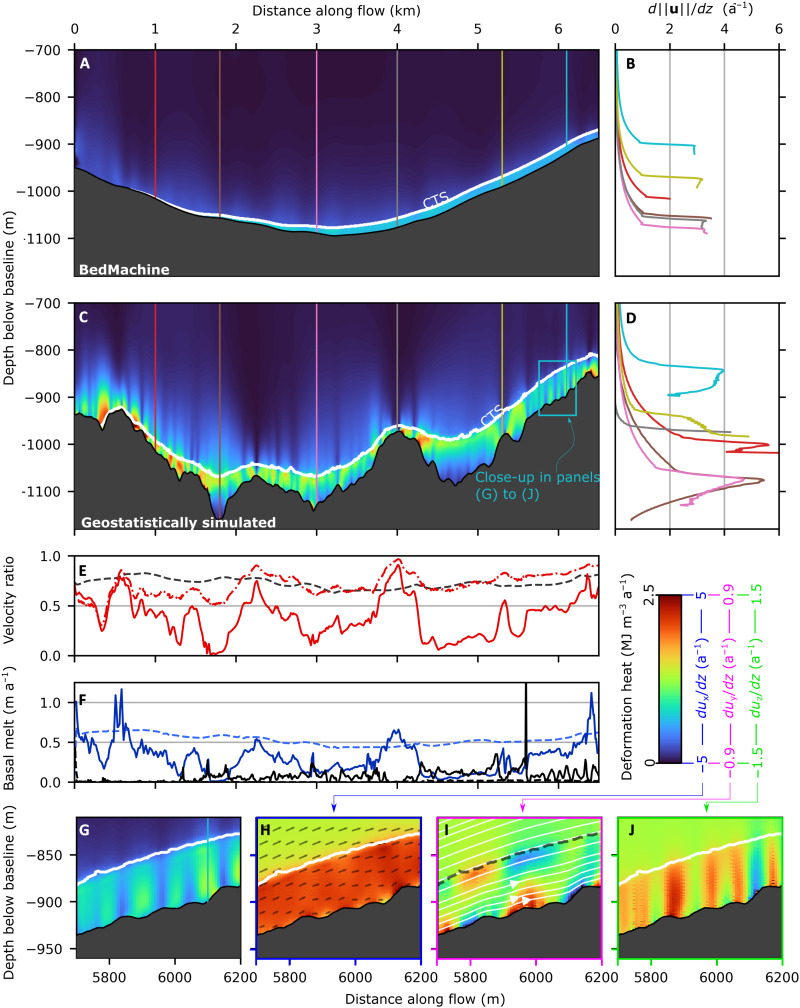
RESPONDER cross section (Sermeq Kujalleq). Cross section in *xz* plane showing deformation heat (product of stress and strain matrices) at *y* = 1300 m (the same *y* value as the transects in Fig. 3) for (**A**) BedMachine topography (run Rbm) and (**C**) geostatistically simulated topography (run Rgb). (**B** and **D**) Deformation rate profiles (change in velocity magnitude with depth) with colors and depths corresponding to the vertical lines in (A) and (C). (**E**) Basal velocity ratio (basal velocity magnitude divided by surface velocity magnitude) for Rbm (black dashed line) and Rgb (red solid line) along the transect and CTS velocity ratio (velocity magnitude at CTS divided by surface velocity magnitude) for Rgb (red dashed-dotted line). (**F**) Basal melt rate in blue and drainage from temperate ice in black for Rbm (dashed line) and Rgb (solid line). Bottommost panels are the close-up in (C) for deformation heat (**G**), change in *x*-oriented velocity with depth (**H**), change in *y*-oriented velocity with depth (**I**), and change in *z*-oriented velocity with depth (**J**). Colored lines from left to right at 1.0, 1.8, 3, 4, 5.3, and 6.1 km. Colored lines in this figure, as well as Figs. 5 and 6, are chosen to highlight interesting deformation behavior. The final 1.5 km is conservatively omitted to avoid the topographic taper and possible outflow boundary condition effects in line with Fig. 7 (see Materials and Methods).

When forced with geostatistically simulated topography (run Rgb; Figs. 3 and 4 and table S1), basal slip rates, internal deformation, and the thickness of the basal temperate layer show great variation across the entire RESPONDER domain, forming a clear contrast to lower variation in the BedMachine topography model output (run Rbm; Fig. 4). With geostatistically simulated topography, the basal temperate ice layer is vertically extensive (>90 m) in topographic depressions, with low basal slip rates (<15 m a^−1^), while fast (~500 m a^−1^) surface velocity shows no local variation. However, the basal temperate ice layer thins markedly (<10 m) over topographic highs, with fast basal slip rates (>500 m a^−1^; Fig. 3, A and B, pink and white rings, respectively). To explore the transition from cold to temperate ice, we track deformation heat and water content changes in flowlines originating ~60 m above the bed 3 km along the *x* axis (Fig. 3E). These show transitions from cold ice with no water content to temperate ice with the maximum allowable water content of 2.5% (see Materials and Methods) over distances as short as ~0.5 to 3 km (Fig. 3C) due to intense internal heat dissipation caused by the movement of ice over topographic obstacles (Fig. 3C, pink and white rings). Deformation heating is notably lower within cold ice regions where ice is stiffer than in the temperate basal layer where ice is much softer (Figs. 2A and 3, C and D, white line). These differences in temperature are initially a result of inflow boundary conditions and then a complex interaction of advection, conduction, and strain heating. Large topographic obstacles also divert ice flow horizontally (Fig. 3F, white ring) and vertically (Fig. 3E), thereby influencing the vertical position of the CTS and rheological properties throughout the domain. The ice sheet (free) surface, with a surface elevation change of 98 m, varies broadly in response to patterns in BedMachine topography, with similar trends across all RESPONDER runs (fig. S2). ParaView output files for all runs across all domains are available in the Supplementary Materials.

Deformation heating profiles for BedMachine (run Rbm) and those from geostatistically simulated topography (run Rgb) are markedly distinct (Fig. 4, A and B). When forced with geostatistically simulated topography, the basal velocity ratio—the basal slip rate divided by the surface velocity—reaches a maximum of 0.91 on a topographic high (Fig. 4C, gray line), where internal deformation drops rapidly above the bed. The basal velocity ratio is smallest within a topographic depression (0.04), where the deformation rate reaches its peak value (5.5 a^−1^) just below the CTS, 90 m above the bed (Fig. 4C, brown line). The CTS velocity ratio—the velocity at the CTS divided by the surface velocity—remains more uniform throughout, peaking over topographic prominences but not dropping below 0.5 after 0.5 km along the transect (Fig. 4E). Profiles also show deformation rates increasing upward (Fig. 4C, blue line), downward (Fig. 4C, yellow line), or even alternating between both (Fig. 4C, red line, 1 km). Strain banding toward the top of the temperate zone is evident in several locations but is not a continuous feature across the entire domain. Distinctive vertical and horizontal banding in deformation heating is seen predominantly within the temperate layer (close-up in Fig. 4, G to J, expanded upon under temperate ice deformation-heating behavior). Basal melting varies with basal sliding (Fig. 4F) but removes basal temperate ice at around 1.3 m km^−1^, making it an important, but not first-order, control on temperate layer thickness in the RESPONDER domain.

The above behavior contrasts the uniform ice motion produced when the model is run with BedMachine topography (Fig. 4A), which gives deformation profiles that are uniform in shape throughout the model domain (Fig. 4B) that broadly conforms with deformation profiles obtained from assumptions of plane strain [e.g., (*16*)]. The thin temperate zone, increasing gradually and uniformly along the transect, accommodates the largest rates of internal deformation (~3 a^−1^) with a monotonic decrease in the ice above. The basal velocity ratio remains high and relatively uniform across the transect with an average value of 0.72.

### Ice motion over a bedrock rise (SAFIRE domain)

**Fig. 5.**
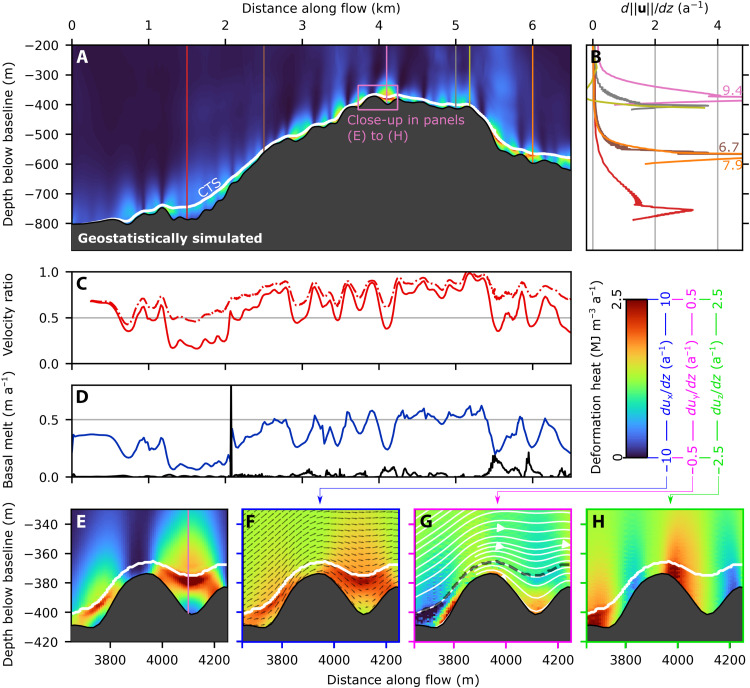
SAFIRE cross section (Sermeq Kujalleq). Cross section in *xz* plane at *y* = 2850 m for (**A**) geostatistically simulated topography (run SAFg). The remainder of the figure (**B**-**H**) follows the same layout as Fig. 4. Colored lines from left to right at 1.5, 2.5, 4.1, 5, 5.18, and 6 km.

At the SAFIRE domain, geostatistically simulated topography (fig. S1) again results in highly variable basal slip rates, ice deformation, and temperate layer thickness (Fig. 5). The temperate layer thins over the large topographic rise in the domain, with a notable increase in basal velocity ratio (Fig. 5A, between red and mauve lines). Over the rise, the basal velocity ratio is high (up to 0.98; Fig. 5A, yellow line) but remains highly variable dropping to a minimum of 0.38 (Fig. 5A, orange line). The CTS velocity ratio still shows obvious variation but is much more uniform across the domain than the basal velocity ratio and only rarely drops below 0.5. Deformation profiles show as much variability as in the RESPONDER simulation; however, rates are higher and more spatially concentrated. Basal melt rates are directly correlated with basal slip rates (Fig. 5, C and D), and drainage from temperate ice is low throughout, increasing on the lee side of the rise to a maximum of 0.22 m a^−1^.

### Ice motion at a land-terminating margin (S5 domain)

**Fig. 6.**
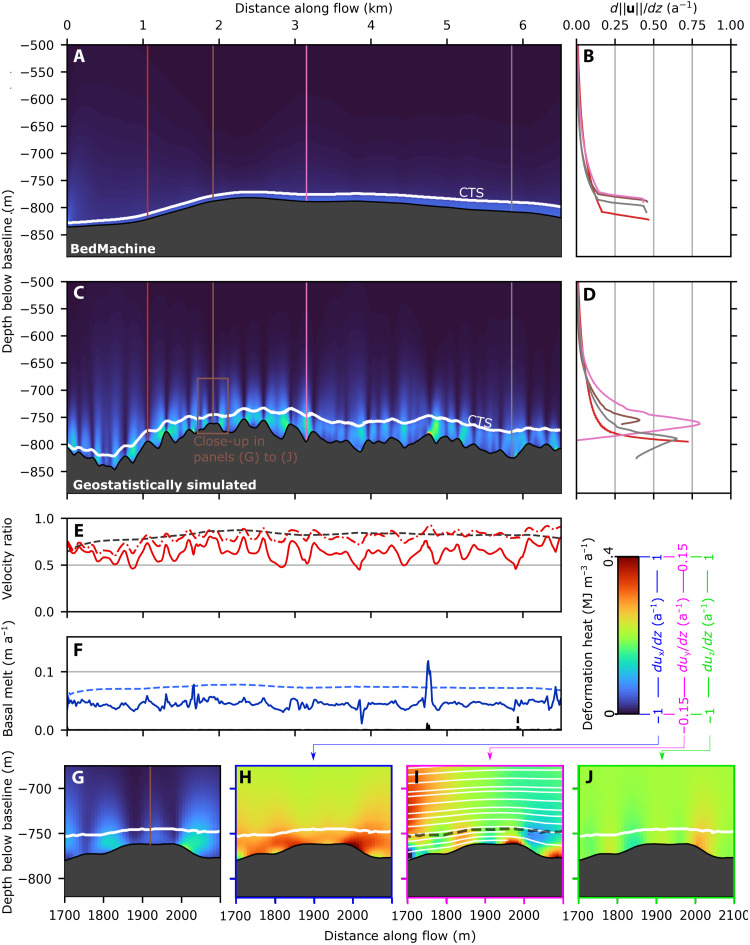
S5 cross section (Isunnguata Sermia). Cross section in *xz* plane at *y* = 2000 m for (**A**) BedMachine topography (run S5bm) and (**B**) geostatistically simulated topography (run S5gb). The remainder of the figure (**C**-**J**) follows the same layout as Fig. 4. Colored lines from left to right at 1.06, 1.92, 3.15, and 5.85 km.

Ice motion and temperate ice behavior at the S5 Isunnguata Sermia site with geostatistically simulated topography are once again notably distinct from other domains, forced by increased short wavelength (<500 m; Fig. 1D) roughness, reduced topographic perturbations at larger (≳2 km) scales, and lower surface velocity. Along the featured transect (Fig. 6C), the basal velocity ratio remains mostly above 0.5 and does not have the same precipitous drops exhibited in the Sermeq Kujalleq domains. High basal velocity ratios (up to 0.81) still occur at topographic prominences, but compared to the Sermeq Kujalleq domains, the CTS velocity ratio shows less departure from the basal velocity ratio, particularly in the first half of the transect where the basal temperate ice layer is thinner (<40 m). Deformation heat is less obviously concentrated toward the top of the temperate zone, instead projecting upward through the CTS and well into the cold ice ~150 m above the bed (close-up in Fig. 6, G to J). Nevertheless, most deformation profiles for geostatistically simulated topography show deformation rates increasing to a maximum just below the CTS, except over some topographic prominences (e.g., Fig. 6D, red line), where deformation decreases monotonically above the bed, as is the case for all deformation profiles for the BedMachine run (Fig. 6B). BedMachine topography produces similar features to the Sermeq Kujalleq BedMachine domains: a gradual temperate layer thickness increase and internal deformation concentrated within the temperate layer.

### Temperate ice deformation-heating behavior

We observe three characteristic patterns of deformation heating, which are covered further in the discussion but introduced briefly here. First, “truncated spires” (e.g., Fig. 4, G to J) refers to places where deformation heating is evident in vertically oriented bands (~50 m across) that terminate abruptly below the CTS. These truncated spires are connected to changes in *x*-oriented velocity with depth (Fig. 4H) and become more frequent as temperate layer thickness increases. This pattern of deformation heating is common in both Sermeq Kujalleq domains but is largely absent in the Isunnguata Sermia S5 domain. Next, “bridges” (e.g., Fig. 5, E to H) are another characteristic feature present where a deformation heating arch below the CTS and above a topographic depression is produced by large changes in *x*-oriented velocity with depth (Fig. 5F). Bridge abutments are produced as the temperate layer vertically extends and depth-averaged velocity decreases on the lee side of a prominence before vertically compressing with an increase in depth-averaged velocity as the trough is exited. Bridges are common in both Sermeq Kujalleq domains but are largely absent in the S5 domain. Last, “cross-cutting spires” or simply “spires” (e.g., Fig. 6, G to J) are similar to truncated spires but protrude some distance (~100 m) above the CTS, gradually reducing in intensity with height. Spires are not only the most common feature in the Isunnguata Sermia domain but also common in the two Sermeq Kujalleq domains in locations where the temperate layer is thinner. We refer to these features without the “deformation-heating” prefix hereafter.

### Domain-wide behavior

**Fig. 7.**
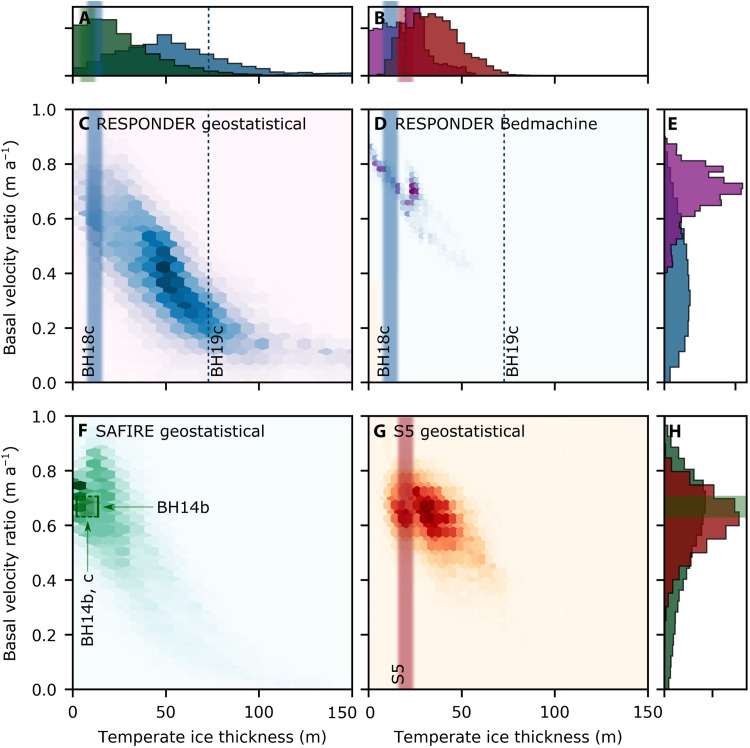
Domain-wide distributions of basal velocity ratio and temperate ice thickness. (**A**) Histograms for temperate ice thickness for RESPONDER and SAFIRE geostatistical domains. (**B**) Histograms for temperate ice thickness for RESPONDER BedMachine and S5 geostatistical domains. (**C**, **D**, **F**, and **G**) Hexbin plots for temperate ice thickness and basal velocity ratios for (C) RESPONDER geostatistical domain (run Rgb; modal bin has 420 counts), (D) RESPONDER BedMachine domain (run Rbm; modal bin has 582 counts), (F) SAFIRE geostatistical domain (run SAFg; modal bin has 755 counts), and (G) S5 geostatistical domain (run S5gb; modal bin has 463 counts). (**E** and **H**) Histograms for basal velocity ratio for RESPONDER geostatistical and BedMachine domains (E) and SAFIRE and S5 geostatistical domains (H). Vertical (A and B) and horizontal (E and H) histogram axes are frequency density. Model data from after 6500 m along flow and within 50 m of lateral boundaries are excluded to reduce the potential influence of boundary conditions and DEM taper, giving a total of 34,677 points per domain (see Materials and Methods). Blue dashed line indicates temperate ice thickness recorded at BH19c, the distributed nature of this measurement means that there is negligible uncertainty. The vertical green, blue, and red bars for BH14c (*25*), BH18c (*64*), and S5 (*26*), respectively, are blurred to indicate uncertainty due to discrete temperature sensor measurements, which may miss the exact location of the CTS. Uncertainty bounds are not provided in the original papers, and we do not attempt to create our own. The green horizontal bar (not blurred) spans the uncertainty range of 0.63 to 0.71 (*25*). The complex basal motion in our model is supported by borehole observations from within each of the three domains (see main text for details).

**Fig. 8.**
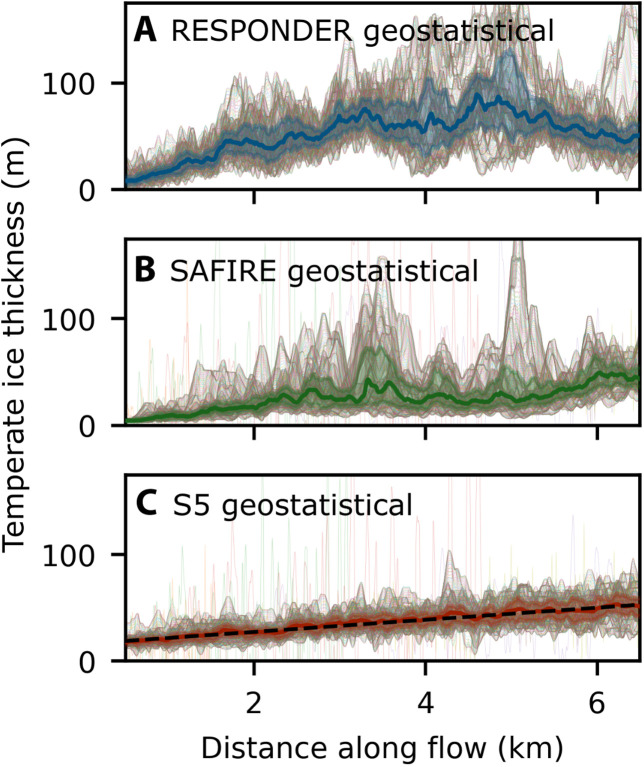
Temperate ice thickness along flow. Temperate ice thickness interpolated into a 5-m orthogonal grid from output triangular mesh. Gray lines are temperate ice thickness for each *y* value along *x* axis–parallel rows. Highlighted area bounds interquartile range obtained for each *y* axis–parallel column, solid colored line is 50% percentile for each *y* axis–parallel column. (**A**) RESPONDER geostatistical domain (run Rgb). (**B**) SAFIRE geostatistical domain (run SAFg). (**C**) S5 geostatistical domain (run S5gb) where the black dashed line is the first-order polynomial fit of the 50% percentile line.

Domain-wide distributions of basal velocity ratio and temperate layer thickness show substantial variation between locations and are further highly dependent on whether BedMachine or geostatistically simulated topography is used (Fig. 7). Runs from Sermeq Kujalleq domains with geostatistically simulated topography exhibit a 1st to 99th percentile range of 0.77 and 0.87 for RESPONDER and SAFIRE, respectively, far exceeding a 1st to 99th percentile range of 0.43 for the RESPONDER BedMachine Run. Temperate ice thickness reaches a maximum modeled value of 189 m in the RESPONDER geostatistical domain compared to only 62 m when BedMachine topography is used. Each hexbin plot for geostatistically simulated topography has a central “hotspot” that clearly varies between domains, with the spread around the hotspot showing a broad linear decrease in basal velocity ratio as temperate layer thickness increases. In contrast to all of the geostatistical runs, the RESPONDER BedMachine hexbin plot (Fig. 7D) is much closer to a line. Use of a different topographic realization produced with the same variogram for RESPONDER does not have a major influence on the shape of the hexbin cloud (fig. S3), indicating that our results are relatively insensitive to the variogram realization used. In runs using geostatistically simulated topography, the temperate ice layer thickness only shows a linear increase along flow in the flatter S5 Isunnguata Sermia domain (Fig. 8C), with average temperate layer thickness increasing to a maximum that coincides with the topographic minima in the RESPONDER domain (Fig. 8A) and increasing overall but with no consistent trend in the SAFIRE domain (Fig. 8B).

Our numerical findings demonstrate that geostatistically realistic topography produces basal slip and internal deformation rates that conform to a broad unimodal distribution. Borehole observations will sample the actual distribution of basal velocity ratio and temperate layer thickness but are too limited in number to be used to produce a statistically “correct” distribution. Nonetheless, our model shows good agreement with borehole observations for all runs with geostatistically simulated topography, matching modal values for temperate ice thickness for SAFIRE and S5. Observed temperate layer thickness falls on either side of the mode for RESPONDER with geostatistically simulated topography, confirming that this spread is present in the GrIS. Given that the observed temperate layer thickness of 73 m at BH19c (*19*) is entirely outside of the distribution produced with BedMachine topography, we argue that realistic topography is a requirement for realistic temperate layer thicknesses and therefore realistic basal motion.

## DISCUSSION

In contrast to the smoothly varying velocity fields produced by most ice sheet models using BedMachine topography, our simulations indicate that basal slip and internal deformation are pervasively heterogeneous, with notable variability over subkilometer distances, and across disparate catchment settings such that even average basal motion properties are substantially different between the two basal topographies considered. In this discussion, we first detail the mechanisms that produce and shape the temperate layer, which has a central role in fast ice motion, before outlining the processes behind the complex basal motion patterns simulated in our model. Both of these aspects are separately compared to borehole observations. Last, while we focus here on descriptions of the complex behavior that emerges from realistic topographic representations, we suggest initial ways to incorporate complex basal motion into numerical parameterizations applicable to large-scale predictive ice sheet models and briefly outline possible directions for future field studies.

### Temperate ice: Formation and observations

Large regions of the GrIS’s bed, including the majority of its margins, are thawed (*31*). This not only facilitates fast ice motion through basal slip but also creates the conditions required for the development of a basal temperate ice layer as we report here. Such a temperate layer has an important but somewhat mysterious role in ice motion as temperate ice is considerably softer than cold ice (*18*, *32*, *33*). This weakness results from liquid water at grain boundaries enhancing diffusion and dislocation creep, dynamic recrystallization, and grain boundary melting [e.g., (*18*, *32*, *34*)] and is particularly important for the basal portions of ice sheets where the largest deviatoric stresses are focused. Temperate ice can be produced by deformation heating above the CTS, with some studies further suggesting the importance of latent heat transfer across the CTS via water in basal crevasses or ice-grain interfaces (*18*, *26*, *35*, *36*). Conversely, all basal heat sources will reduce temperate layer thickness through basal melt as the positive upward temperature gradient defined by the Clausius-Clapeyron slope operates as a thermal barrier. Basal heat sources include viscous heat dissipation in flowing subglacial water (*37*), geothermal heat flux, and frictional heat from sliding. While these theoretical underpinnings are well established (*26*, *35*, *38*, *39*), we show that realistic basal topography is the crucial additional component required to explain borehole observations of complex variation in temperate layer thickness across the GrIS (*16*, *19*, *20*, *25*, *26*, *40*, *41*).

At the Isunnguata Sermia S5 domain, the interquartile thickness (obtained across width) of the basal temperate layer increases at 4.7 to 6.7 m km^−1^ when forced with geostatistically simulated topography (Fig. 8C). While high variability in the spatial rate of change of temperate layer thickness is to be expected (discussed below), our modeled temperate layer growth rate is compatible with Isunnguata Sermia borehole observations where the temperate layer thickness increases at a rate equivalent to ~4.3 m km^−1^ between site S5 and site S4, located 18.4-km downstream in the direction of flow (Fig. 1B) (*26*). As we do not include water transport in our model, our results indicate that roughness-controlled deformation heating alone is sufficient to produce temperate ice at observed rates.

The importance of high-resolution realizations of basal topography is further evident when comparing model outputs forced with geostatistically simulated topography and BedMachine topography, respectively. When topography is smooth (BedMachine run), deformation heat is concentrated within a relatively thin temperate zone and is therefore contributing to internal melt of the temperate layer, not temperate layer growth. A greater basal velocity ratio additionally means that basal frictional heating, and therefore basal melt, is increased relative to deformation heat above the CTS. In contrast, perturbations ~200 m across present in geostatistically simulated topography result in ubiquitous spires protruding through the CTS (Fig. 6, G to J), which effectively warm cold ice to the point that it becomes temperate. These spires begin to truncate at the CTS as temperate ice thickness increases (Fig. 4C)—which can be explained by the temperate layer now being sufficiently thick as to accommodate a greater proportion of internal deformation—indicating that the temperate layer thickness may not continue to increase linearly indefinitely. This nonlinearity, as well as the situation of S3 on a topographic rise, may explain why the observed increase in the temperate layer at Isunnguata Sermia is smaller than predicted in the S5 model domain in isolation. Overall, we view deformation heating above the CTS as a simpler and more general explanation for temperate layer growth than the hydrological mechanism hypothesized by (*26*, *36*), which requires pervasive basal crevasses or intergranular water transport, both of which lack a clear observational basis.

Localized shear banding and bridges between topographic prominences are further distinctive features of the temperate layer forming in our model. These features are most easily understood by considering their development as the model approaches steady state (fig. S4). As ice slips through a topographic depression, it is physically unable to do so without deformation heating increasing the specific enthalpy of the ice locally and downstream and therefore decreasing its viscosity (Fig. 2A)—a system that stabilizes at the point where deformation heating balances conductive and advective heat losses (*38*). Bridges thereby connect topographic highs where basal slip is high sensu stricto with concentrated internal deformation toward the top of the temperate layer, which fills the intervening trough, enabling rapid movement of cold ice over comparatively stagnant temperate ice below. These bridges contribute to some heating above the CTS and occur far more frequently in our two fast-moving domains (RESPONDER and SAFIRE; Figs. 4C and 5A), which may be explained by a velocity threshold controlling the transition between these modes.

Bridges and (truncated) spires help to explain the complicated temperate ice variation modeled and observed at Sermeq Kujalleq. Spires that protrude through the CTS in the RESPONDER domain (Fig. 4C) increase the temperate layer thickness to a maximum across-flow average of 100 m after 5 km (Fig. 8), beyond which bridges and truncated spires dominate, and the rate of temperate layer growth decreases. Convex large-scale (≳2 km) topography further prompts vertical compression and acceleration of the entire ice column, reducing the absolute thickness of the temperate layer and concentrating more deformation below the CTS. This variation is supported by, and further helps to explain, borehole observations of temperate ice. A 73-m-thick temperate layer observed from borehole BH19c (*19*) within the RESPONDER site likely indicates a subglacial topographic depression, while a much smaller temperate layer thickness (<20 m) only a few kilometers away (but still within the RESPONDER domain; Fig. 1A) (*25*) is more likely to sample less temperate ice above a topographic prominence. At SAFIRE, notable modeled temperate-zone thinning over a notable bedrock rise is compatible with borehole observations from this site showing either a very thin (<8 m) or absent temperate layer (*25*). Here, the convex large-scale topography increases basal-slip and hence reduces internal deformation heating. As with RESPONDER, thinning of the entire ice column over a rise will also locally reduce the absolute thickness of the temperate layer.

Our numerical model also aids interpretation of other GrIS borehole observations. At a site at 12.75 km from the ~20-m-thick temperate ice layer at S5 (Isunnguata Sermia) and ~1 km south of the main flow line (hereafter IS2015; Fig. 1B), Maier *et al.* (*20*) observe no substantial temperate ice and a high basal-velocity ratio, while a ~100-m-thick layer of temperate ice was observed just 8 km further along flow at sites S4 and S3 (*26*). We suggest that this spatial variability is connected to the topographic rise on which IS2015 was located, which can compress and accelerate the overlying ice in a similar manner to modeled ice motion over the SAFIRE rise beneath Sermeq Kujalleq. However, we also emphasize that stochastic spatial variation in temperate layer thickness, related to local (100 s of m) topographic relief, may play an additional role in intersite variability. This local variation may further explain observations near Swiss Camp, where temperate layer thickness decreased from ~40 to ~20 m over 10 km along flow (*16*), which could reflect natural variability as indicated by individual temperate layer thickness profiles in Fig. 8. Last, our findings fully support the existence of an inferred extremely thick (>300 m) basal temperate layer in the deeply eroded basal trough of Sermeq Kujalleq (Jakobshavn Isbræ) formed largely by vertical ice extension (*17*) and offer further avenues to test its importance in fast ice motion. Overall, considerations from our results and from borehole records indicate that large-scale topographic variations (e.g., rises and saddles) control broad patterns of temperate layer thickness, while geostatistically simulated topography is central to the formation of temperate ice and to intermediate-scale (≥25 m, ≲4 km) variations in its thickness.

These results emphasize not only the importance of temperate ice but also the paucity of information regarding temperate ice at micro- and macroscales, particularly for the GrIS. Recent laboratory studies on temperate ice are limited [e.g., (*34*, *42*)], and the parameter space for temperate ice flow is relatively unconstrained [e.g., (*18*, *34*, *35*, *43*)], with temperate ice modeling studies mainly directed toward Antarctic shear margins. Although we do not include anisotropy, the rheology of temperate ice in our model is at the most viscous end of plausible values (see Materials and Methods), with less viscous formulations having the potential to further increase the deformation accommodated within the temperate layer. Alternative drainage formulations for temperate ice may also influence rheological properties and therefore temperate layer behavior (*35*). While our results reproduce key field observations and provide a framework for understanding temperate ice formation and behavior, further work is urgently required to constrain appropriate parameters and parameterizations for temperate ice.

### Complex basal motion: Simulations and observations

The model behavior outlined thus far is supported by, and provides an explanation for, the broad spectrum of ice-motion behavior revealed in GrIS borehole records—in addition to the temperate layer thickness variability outlined above. At RESPONDER, damage to a fiber-optic cable at the center of the modeling domain indicated a clear increase in ice deformation toward the top of the temperate ice layer that could not be explained by existing theories (*19*). Our model reproduces this strain behavior when a thicker temperate ice layer occupies a local or broad topographic depression (Fig. 3, C and D brown, pink, and blue lines), showing that this deformation heterogeneity is an intrinsic part of GrIS ice motion. As this behavior is reproduced with a near-constant rate factor within the modeled temperate layer—a result of uniform water saturation shortly below the CTS—our results further indicate that varying ice rheology is not a prerequisite for variable temperate layer deformation, as hypothesized in (*19*), but rather realistic bed topography is. No deformation profile from the BedMachine domain reproduces the fiber-optic cable damage pattern, further indicating that BedMachine topography will not produce realistic ice-motion behavior at intermediate scales (≲4 km).

SAFIRE domain model results also show similarly good agreement with observations. Here, Doyle *et al.* (*25*) obtain a basal velocity ratio of 0.63 to 0.71 and a temperate layer thickness of up to 8 m, very close to the modal bin of Fig. 7F (sliding ratio of 0.75 and temperate layer thickness of 4 m). Further, the borehole tilt sensor deformation peak of 1.8 a^−1^ 19 m above the ice sheet base (*25*) is entirely compatible with our modeled deformation rate increases directly (<50 m) above the base (e.g., Fig. 5, A and B, orange and pink lines). Modeled deformation rates change rapidly with distance above the bed, thus 19-m-above-the-bed sensor of Doyle *et al.* (*25*) may not necessarily represent the maximum rate of deformation within the borehole, which could feasibly continue increasing beyond 1.8 a^−1^ following the gradient between the two lowermost tilt sensors.

As our modeling results indicate that strain bands are spatially limited yet carry a large proportion of ice motion, this implies that basal slip estimates gathered from discrete sensors may be more uncertain and variable than appreciated so far. Linear interpolation of discretely spaced sensors omitting spatially concentrated strain banding may furthermore underestimate internal deformation and therefore overestimate basal velocity ratios. Another bias comes from the fact that most boreholes in Greenland have been drilled over bedrock highs for practical reasons. Together, the SAFIRE and RESPONDER model runs contrast the uniform glacier motion produced in previous Sermeq Kujalleq models forced with smooth BedMachine topography [e.g., (*14*)] and suggest that the complex basal motion is the norm rather than the exception in marine-terminating outlet glacier settings.

Complex basal motion also helps to explain observations from the slower-moving land-terminating ice sheet margin at Isunnguata Sermia that may at first appear contradictory. Site IS2015, just 12.75 km from S5 (Fig. 1B, outlined under temperate ice formation) (*20*), has a measured basal velocity ratio ranging from 90.6 to 99.7% and no substantial temperate layer. These basal velocity ratio and temperate layer thickness are distinct from (although not completely incompatible with) the distribution modeled at S5. However, we emphasize that the difference between the closely spaced RESPONDER and SAFIRE domains indicates that a high level of variation forced by different long-wavelength topography is not unusual and should be expected. Our analysis of temperate ice formation along the S5 flowline further suggests that if sliding rates are pervasively as high elsewhere in the Isunnguata Sermia catchment as those at IS2015, then insufficient deformation heat will occur to prompt the very large observed temperate layers at S1 to S4 (*26*). Therefore, while it has been hypothesized that sliding is the most important component of ice motion in land-terminating sectors of the GrIS (*20*), our model shows that sliding dominance is confined to topographic high points and is not a general condition of motion across the GrIS.

### Complex basal motion: Application to large-scale models

Our process-based understanding of basal motion at small (<25 m) scales has progressed significantly in recent years. Laboratory work for ice overlying deformable sediments (*22*) and 3D numerical modeling of glacier slip over hard beds with water-filled cavities (*23*) both suggest that basal traction conforms to a regularized Coulomb relationship: Slip resistance first increases with slip velocity before reaching a threshold velocity where till shears at its rate-independent yield strength or cavity dimensions stabilize (Fig. 2B). However, while some large-scale studies support the above experiments [e.g., (*8*, *10*)], this is not universal, with other studies suggesting a Weertman-type slip relationship of ice motion, where basal traction increases indefinitely with increasing basal velocity (*44*, *45*). The selected basal slip relationship is then used in an inversion procedure over smooth BedMachine topography for basal traction parameters that are typically assumed to be time invariant.

The above contention surrounding appropriate parameterizations has important ramifications. The choice of basal slip relationship significantly alters ice sheet model predictions [e.g., (*8*, *9*, *46*)], and “deep” process uncertainty in ice sheet models is a key concern in the most recent Intergovernmental Panel on Climate Change report (*3*). We propose that expanding our conception of ice sheet motion to include intermediate-scale flow variability (≥25 m, ≲4 km), as well as the complexity under topography and basal conditions clearly evident from observations beneath past [e.g., (*47*)] and present [e.g., (*48*–*50*)] ice sheets, offers a route forward. Parameters derived from inverse methods for heuristically applied basal slip relationships (including Weertman or regularized Coulomb) over smooth BedMachine topography can implicitly account for the complex ice motion described here. However, while parameters from inversions may reproduce observed velocities well, divergence between predicted and actual ice sheet behavior is likely to increase over model time if the form of the basal motion relationship is an incorrect representation of intermediate-scale basal motion processes and does not account for temporal changes under motion conditions (*51*). For example, a local increase in temperate layer thickness may reduce the basal velocity ratio but increase ice discharge overall as local resistance to flow decreases.

Incorporating the process-based understanding we have generated here into a parameterization that explicitly accounts for basal motion over realistic topography provides a potential solution. This is achievable by determining how the area-integrated basal traction varies with the area-integrated basal velocity over areas representative of grid or element sizes of large scale models, when the domain slope, and therefore driving stress, is incrementally increased [similar in approach to (*23*)]. The sensitivity of this relationship to (i) roughness and morphology of the basal topography, (ii) thickness of the temperate layer, and (iii) the dependence of the basal traction relationship on the size of the area considered can then be assessed. To address point (i), future work is needed to develop a subglacial roughness measure that can be used as a spatially invariant parameter in a basal motion relationship. Variogram parameters may suffice for this purpose. Consistency across our simulations using the same variogram (fig. S3) also means that the basal motion characteristics could be determined from the variogram alone, without requiring simulation of topography. In terms of point (ii), a quantitative assessment of temperate layer thickness development and its effect on ice flow sensitivity to slope changes would allow the temperate layer’s influence on resistance to ice motion to be isolated and ultimately parameterized. Last, point (iii) may explain why simple sliding relations such as by Weertman (*52*) are still appropriate at a scale of multiple kilometers [e.g., (*44*)] despite a lack of incorporation of key small-scale processes (*53*). We outline some further geological considerations in Supplementary Text.

Our proposed parameterization approach may also mean that the complex basal motion we have identified at intermediate scales can be incorporated in large-scale ice sheet models with only minor modifications and without requiring increased model resolution. Development and implementation of such a parameterized basal motion relationship will increase confidence that predictive ice sheet models are accurately representing the complex reality of ice sheet motion and may therefore improve the accuracy of sea level rise predictions.

### Outlook

Our results show that while the basal velocity ratio and temperate layer thickness can vary across a small region (~0.25 km^2^) and may mimic catchment-scale results, most small regions in isolation will not be representative of basal motion at larger scales. A focus in field studies on coarser (≳1 km) borehole arrays covering a wider range of topographic features may therefore enable more accurate characterization of ice motion variability. Separately, as temperate layer thickness variation is directly influenced by deformation heating within the ice and hence the basal velocity ratio, intensive borehole- and radar-based investigation across a domain similar in size to the ones used here would allow improved estimates of parameters by fitting model data to observations.

Overall, our results indicate unavoidable complexity in descriptions of ice sheet motion. We provide a unified explanation for borehole observations of spatial variability in basal temperate ice thickness and basal velocity ratio and for down-borehole variability in deformation rates. In sum, we hope that these advances in understanding will facilitate the development of improved representations of ice sheet motion and hence more accurate predictions of sea level rise.

## MATERIALS AND METHODS

### Numerical modeling

We model ice flow in rectangular 8 km (along flow) by 4 km (across flow) domains oriented along flow where the *x*, *y*, and *z* axes define length, width, and depth, respectively (Fig. 1F). This allows a high mesh resolution and a focus on basal-motion processes, without requiring modeling of an entire glacier catchment. To obtain realistic boundary conditions for our model domains, we first use time-evolving runs with periodic inflow-outflow conditions (Fig. 2C) and a periodic free-surface runs (FS runs) to determine the gravity vector orientation (or slope) needed to approximate satellite-derived glacier velocities characterizing each domain. We then obtain a free-surface digital elevation model (DEM), surface pressure field, and inflow boundary conditions for the velocity vector components and pressure. We use these derived quantities as fixed boundary conditions and keep the orientation of the gravity vector as calculated in the FS runs for the final thermomechanically coupled runs (TC runs) in which the enthalpy and velocity fields are allowed to evolve until steady-state convergence is reached. See “Free-surface runs” and “Thermomechanically coupled runs” sections, respectively, for full boundary condition statements. TC runs are not compatible with periodic boundary condition, as it is unphysical for the enthalpy field and hence rheological characteristics at the outflow boundary to match the inflow boundary when the temperature field evolves along flow. We do not display or use the final 1.5 km of our domains in Figs. 4 to 8, as we conservatively omit this to account for any possible boundary condition interference and for most of the 1.6 km topographic taper used to make the basal topography periodic in the along-flow direction (see “Geostatistical DEM simulations” section). For similar boundary condition considerations, we also omit the first and last 50 m across flow in analysis for Figs. 7 and 8.

We use the Elmer/Ice (version 9.0) finite element modeling package (*54*) on GNU/Linux with 191.9-GB total memory and 18 @2.20-GHz processor partitions for all runs. FS runs take ~5 days, and TC runs take ~12 hours. A triangular mesh with representative edge length of 25 m and ~124,119 triangular elements is made with Gmsh and vertically extruded using the Elmer/Ice StructuredMeshMapper (*55*), with vertical layer spacing decreasing toward the base. Further increasing the spatial resolution does not meaningfully alter model output (fig. S5). FS runs use 25 vertical layers to reduce computation time, increased to 42 for TC runs, giving a lowermost cell thickness of 1.6 m for an ice column of 1 km (fig. S6). Domains are centered about a borehole location, with the basal topography normalized such that the average DEM value is equivalent to the negative of the thickness obtained by the central borehole(s), giving an initially flat surface with *z* coordinate of 0 m. To maintain inflow-outflow boundaries at the same *z* coordinates, the SAFIRE domain is additionally detrended to remove an average slope of 2.7°. RESPONDER and S5 cover areas with small or negligible inflow-outflow displacement in the context of across-flow topographic variation at these boundaries, so no detrending is applied. Table S1 provides details on specific run setups. Table S2 provides parameter and constant values.

We solve the standard Stokes equations governing ice flow

∇⋅u=0(conservation of mass) (1)

∇⋅τ−∇⋅p=−ρg(balance of momentum) (2)

where **u** (m a^−1^) is the ice velocity, **τ** (MPa) is the deviatoric stress tensor, *p* (MPa) is the ice pressure, ρ (MPa m^−2^ a^−2^) is the ice density (assumed constant, with no adjustment for water content) and **g** (m a^−2^) is the gravitational acceleration vector. The slope, θ (in degrees), is set by assigning **g** = [*g* sin (θ),0, − *g* cos (θ)], where *g*= 9.81 m s^−2^, to remove the requirement for vertical displacement of periodic inflow-outflow boundaries. Boundary conditions are specified for FS and TC runs separately. Stress is related to strain using the Nye-Glen isotropic flow law (*56*, *57*),

$$\dot{\text{ϵ}}=A\tau_{e}^{n-1}\text{τ}$$ (3)

where $\dot{\text{ϵ}}$ with ${\dot{\text{ϵ}}}_{ij}=\frac{1}{2}\left(\frac{\partial u_{i}}{\partial x_{j}}+\frac{\partial u_{j}}{\partial x_{i}}\right)$ (a^−1^) is the strain rate tensor, $\tau_{e}^{2}=\frac{1}{2}\mathrm{tr}({\text{τ}}^{2})$ (MPa) is the effective stress in the ice, *n* is the flow exponent assumed to equal 3, and *A* (MPa^−3^ a^−1^) is the creep parameter. *A* is calculated from the homologous temperature, *T*_h_ (in kelvin), if below the pressure-dependent melting point, *T*_m_, or water content, ω (proportion), if above as

$$A=\{\begin{array}{c} A_{1}\exp\left(\frac{Q_{1}}{RT_{h}}\right),T_{h}\leq T_{\lim}\\ A_{2}\exp\left(\frac{Q_{2}}{RT_{h}}\right),T_{\lim}<T_{h}<T_{m}\\ (W_{1}+W_{2}\omega \times 100)W_{3},T_{h}\geq T_{m},\omega <\omega_{\mathrm{llm}}\\ A_{\max},\omega \geq \omega_{\lim} \end{array}$$ (4)

where *T*_m_(*p*) = *T*_tr_ − γ(*p* − *p*_tr_), γ (K MPa^−1^) is the Clausius-Clapeyron constant, *T*_tr_ is the triple point water temperature, and *p*_tr_ is the triple point water pressure. *A*_1_ and *A*_2_ (MPa a^−1^) are rate factors, and *Q*_1_ and *Q*_2_ (J mol^−1^) are activation energies for *T* ≤ *T*_lim_ and *T*_lim_ < *T* < *T*_m_, respectively, where *T*_lim_= 263.2 K is the limit temperature. *R* (J mol^−1^ K^−1^) is the gas constant, and *W*_1_, *W*_2_, and *W*_3_ (all in MPa a^−1^) are water viscosity factors, with default values taken from the linear fit of Duval (*32*) adapted by Haseloff *et al.* (*43*) for water contents up to 0.7 ± 0.1% under tertiary creep. We hold *W*_1_, *W*_2_, and *W*_3_ constants for all model runs and set a conservative limit for ω_lim_ of 0.6%, as Adams *et al.* (*34*) propose that *A* does not increase between water contents of 0.6 to 2% following experiments conducted under secondary creep. Once ω_lim_ is exceeded, *A* = *A*_max_, limiting the rate factor of temperate ice. Figure 2A shows the increase in *A* with temperature and then water content as used in our model.

Specific enthalpy, *H* (J kg^−1^), is used as the state variable with the Elmer/Ice EnthalpySolver (*58*) and is related to *T* and ω as

$$H(T,\omega )=\{\begin{array}{c} \frac{1}{2}C_{a}(T^{2}-{T_{\mathrm{ref}}}^{2})+C_{b}(T-T_{\mathrm{ref}}),H<H_{m}(p)\\ \omega L+H_{m},H\geq H_{m}(p) \end{array}$$ (5)

where *C*_a_ (J kg^−1^ K^−2^) and *C*_b_ (J kg^−1^ K^−1^) are enthalpy heat capacity constants, *L* (J kg^-1^) is the latent heat capacity of ice, $H_{m}(p)=\frac{1}{2}C_{a}[T_{m}{\left(p\right)}^{2}-{T_{\mathrm{ref}}}^{2}]+C_{b}[T_{m}(p)-T_{\mathrm{ref}}]$ is the specific enthalpy at the pressure-dependent melting point, and *T*_ref_ = 200 K is the reference temperature.

We follow the small-scale physically derived regularized Coulomb relationship of (*23*), which appropriately accounts for sliding within our mesh elements with representative edge length of 25 m. Basal traction, τ_b_ (MPa), is given as

$$\tau_{b}=CN_{e}{\left(\frac{{u_{b}}^{-n+1}}{u_{b}+A_{s}C^{n}N_{e}^{n}}\right)}^{\frac{1}{n}}u_{b}$$ (6)

where *C* (dimensionless) is a parameter that depends on basal morphology and cannot be readily estimated from irregular topographies but must be less than the maximum up-slope gradient of the bed (*23*), *u*_b_ is the basal velocity (m a^−1^) tangential to the ice-bed interface, and *n* = 3 is the same exponent used in the flow relationship. *A*_s_ (m a^−1^ MPa^−3^) depends on ice rheology and morphology of the bed and is used in the case of hard-bed sliding with no cavitation (*52*), and *N*_e_ = *p*_i_ − *p*_w_ (MPa) is the effective pressure at the bed where *p*_i_ (MPa) is the ice overburden pressure and *p*_w_ (MPa) is the subglacial water pressure. Helanow *et al.* (*23*) provide six values for *A*_s_ and *C* based on representative element-area DEMs obtained from uncrewed aerial-vehicle surveys on bedrock surfaces recently exposed by glacier recession. We take the average of these six values for each of *A*_s_ and *C* as constant for all runs rather than apply a basal-traction inversion procedure that would require inherent assumptions about ice deformation. *N*_e_ is then varied as the only free parameter controlling basal traction, although we note that this has a similar effect to varying *C*.

We make the simplifying assumption that *N*_e_ is related to the overburden pressure alone (thereby omitting a more complicated subglacial drainage system) via a proportionality parameter, *O* (dimensionless), as *N*_e_ = − ρ*g_z_O*(*s* − *b*) where *b* (m) is the elevation of the glacier base and *s* (m) is the surface elevation. An *O* of 1 is equivalent to a water pressure of 0, while an *O* of 0 means water pressure effectively balances ice overburden pressure. However, the upper limit of basal traction tangential to the bed under Eq. 6 is τ_b_max__ = *N*_e_*C*, which can lead to model instability if the areally averaged basal traction cannot support the driving stress, τ_d_ = ρ*g_x_*(*s* − *b*), across the domain. Setting τ_b_max__ ≥ τ_d_ and expanding *g_x_* and *g_z_* then give the inequality

$$O\geq \frac{F\;\tan(\theta )}{C}$$ (7)

where *F* is a parameter we introduce to account for the resistive influence of intermediate-scale topographic obstacles, or lack thereof (further description in Supplementary Text). For the range of slopes and *F* values covered and *C*= 0.1617, this gives a range in *O* from 0.0874 to 0.240, comparable to values in other studies (*37*, *59*). Increasing the proportion of driving stress supported by the maximum basal traction value slightly shifts the basal-velocity-ratio distribution toward lower values, with a new mode of 0.28 (fig. S3). θ is then altered in 0.05° increments (with concomitant change in *O*) to obtain the best match between modeled surface velocity and satellite measurements. Surface slopes in the regions studied are 1° to 2° with variation in **g** away from the long-wavelength borehole site value expected, as **g** in our model will also be accounting for longitudinal and transverse stresses in the ice. Altering the basal topography slope will slightly alter the relative angle of obstacle stoss and lee sides, but we view this change as small relative to the absolute angle of these obstacles (up to 40°).

### Free-surface runs

In FS runs, enthalpy and *A* are calculated as a function of normalized depth, $D=\frac{d}{s-b}$, where *d* (m) is depth, as

$$H=E_{a}D^{2}+E_{b}D+E_{c}$$ (8)

where *E_a_*, *E_b_*, and *E_c_* are quadratic curve parameters. *E_a_*, *E_b_*, and *E_c_* are obtained via a second-order polynomial fitting procedure (MATLAB polyfit) of the borehole temperature record at the center of the domain, converted to enthalpy with minor adjustments to only remove the temperate ice layer (fig. S7). This approach ensures consistent rheology at the periodic inflow-outflow boundaries. These profiles are also used for the input enthalpy field in TC runs.

The free surface is computed with the Elmer/Ice FreeSurfaceSolver as

$$\frac{\partial s}{\partial t}+u_{x}\frac{\partial s}{\partial x}+u_{y}\frac{\partial s}{\partial y}=u_{z}$$ (9)

where *u_x_*, *u_y_*, and *u_z_* are components of the surface velocity vector **u**. No accumulation or ablation is accounted for in FS runs, as this would require a corresponding removal of mass from elsewhere in the model in order for mass to remain constant. Outstanding FS boundary conditions are

u⋅n=0onz=b(impenetrability condition at base) (10A)

$$\;\begin{array}{rl} & u\cdot n=0\;\mathrm{on}\;y=0\;\mathrm{and}\;y=y_{\max}\\ & \left(\text{impenetrability condition on lateral sides}\right) \end{array}$$ (10B)

$$\;\begin{array}{rl} & t_{l}\cdot \text{σ}n=0\;\mathrm{on}\;y=0\;\mathrm{and}\;y=y_{\max}\\ & \left(\text{free sliding tangential to boundary}\right) \end{array}$$ (10C)

where **σ** is the Cauchy stress tensor, **n** is the normal vector, and **t***_l_* (*l*= 1, 2) are tangent vectors at the specified surface, with tangential stress at the bed specified in Eq. 6 and a stress-free surface. We note that the free-slip no-flux lateral boundary conditions here and in TC runs (Eqs. 15C and 15D) effectively create constrained flow within the domain. While we selected sites for relatively low convergent flow (Fig. 1, A and B) given the marginal setting, we note that this may become more important where convergent flow is greater but could be remedied with converging lateral domain boundaries. The periodic inflow-outflow boundaries in FS runs, in addition to channel flow, further means that the modeled ice surface will only reflect the modeled basal topography, not the broader topographic changes at Sermeq Kujalleq and Isunnguata Sermia. The time step is set to 0.015 a, and the simulation is stopped when the maximum and minimum surface show only minor variation (fig. S8). The free-surface DEM, surface pressure field, and inflow pressure and velocity fields are then extracted and reprojected as boundary conditions onto the TC mesh.

### Thermomechanically coupled runs

The specific enthalpy field is set at the inflow boundary based on depth and the central borehole record following Eq. 8 and allowed to freely evolve until a steady state is reached and is calculated as

$$\rho \left(\frac{\partial H}{\partial t}+u\cdot \nabla H\right)=\nabla (\kappa \nabla H)+\mathrm{tr}(\text{τ}\dot{\text{ϵ}})$$ (11)

where $\mathrm{tr}(\text{τ}\dot{\text{ϵ}})$ is the strain heating term. κ (kg m^−1^ s^−1^) is the enthalpy diffusivity defined as

$$\kappa =\{\begin{array}{c} \kappa_{c},H<H_{m}(p)\\ \kappa_{t},H\geq H_{m}(p) \end{array}$$ (12)

where κ_c_ and κ_t_ are enthalpy diffusivities for cold and temperate ice, respectively, meaning that water movement within the temperate ice is assumed to be a diffusive process. *H* is limited by ω_max_ set at 2.5%, around the level of field observations of water content [(*60*) and references therein]. However, as we limit increases in *A* to water contents of 0.6%, we note that a greater maximum water content value only acts to increase water-content gradient, and hence, enthalpy transfers within the temperate zone and across the cold-temperate transition zone, although κ_t_ is an order of magnitude lower than κ_c_. Drainage is treated simply by setting an upper specific enthalpy limit equivalent to ω_max_ with drainage assumed to occur instantaneously above this threshold. Vertically integrated drainage volumes, *D*_v_ (m^−3^ a^−1^), are then obtained from specific enthalpy loads, *H*_loads_, and element weighting, *H*_weights_, as

$$D_{v}=\int_{b}^{\mathrm{CTS}}\frac{H_{\mathrm{loads}}}{H_{\mathrm{weights}}}dz$$ (13)

where CTS is the *z* coordinate of the CTS. More advanced drainage formulations exist (*35*), but their implementation is beyond the scope of this paper.

For the purposes of basal sliding, we make no adjustment for the small sections where basal temperatures are below the pressure melting point, effectively assuming that the bed is sufficiently well-hydrologically connected to provide water to these regions for freeze-on. Considerations for basal freeze-on rates required to pin the basal temperature at the pressure melting point are presented in the Supplementary Materials, showing that the water required is negligible in context. The basal mass balance, *M*_b_ (kg m^−2^ a^−1^), is calculated as

$$M_{b}=\frac{1}{H}(F_{b}+Q_{b}+G_{b}\;\cdot \;n-q\;\cdot \;n)$$ (14)

where *F*_b_ = *u*_b_τ_b_ is the frictional heating at the bed, **G**_b_ is the geothermal heat flux, q = −*k* ∇ *T* is the energy flux into the ice where *k* is the thermal conductivity of the ice, and *Q*_b_ is the rate at which hydrological storage and transport mechanisms deliver latent heat to the base of the ice that we set as 0 across all of our model domains. When a temperate layer is present, q is a small negative value (flux directed toward the base) determined by the local pressure gradient. If no temperate layer is present, then q is a small positive value (directed away from the base). Comparisons of inflowing and outflowing ice volumes for the RESPONDER BedMachine run, where mass loss is greatest, show that basal melting has a negligible effect on overall ice flow volumes (0.3%). This leaves outstanding TC boundary conditions as

$$\;\begin{array}{rl} & u\;\cdot n=0\;\mathrm{on}\;z=s\\ & \left(\text{impenetrability condition at fixed surface}\right) \end{array}$$ (15A)

σn=0onz=s(stress-free surface) (15B)

$$\;\begin{array}{rl} & u\;\cdot n=0\;\mathrm{on}\;y=0\;\mathrm{and}\;y=y_{\max}\\ & \left(\text{impenetrability condition on lateral sides}\right) \end{array}$$ (15C)

$$\;\begin{array}{rl} & t_{l}\;\cdot \sigma n=0\;\mathrm{on}\;y=0\;\mathrm{and}\;y=y_{\max}\\ & \left(\text{free sliding tangential to boundary}\right) \end{array}$$ (15D)

$$p=\rho g_{x}d\;\mathrm{on}\;x=x_{\max}\;(\text{hydrostatic pressure at outflow})$$ (15E)

$$\;u\;\cdot n=M_{b}\;\mathrm{on}\;z=b\;(\text{basal melt condition})$$ (15F)

Elmer/Ice solver input files and postprocessing scripts are available in the Supplementary Materials.

### Geostatistical DEM simulations

We use conditional geostatistical simulations (*27*) to produce DEMs for each site that (i) match basal topography from radar flight lines that cross each domain, (ii) reproduce the roughness exhibited in radar flight line profiles (Fig. 1), and (iii) retain the long-wavelength (≳2 km) features of BedMachine. To create the most direct comparison with BedMachine topography (*12*), we simulate topography by adding roughness to BedMachine based on the characteristics of the residual between BedMachine and radar measurements. It is customary to simulate multiple realizations to quantify uncertainty [e.g. (*30*)]; however, no great variation in the form of the hexbin cloud for the RESPONDER domain is produced when the model is forced with topography from the second topographic realizations of this region (fig. S3), so only one realization is used for the SAFIRE and S5 domains. Two realizations are used for the RESPONDER domain for a sensitivity analysis.

The simulation is carried out in the following steps. First, the residuals between BedMachine v3 and CReSIS radar measurements [from 1993 to 2017) (*61*) are calculated. We included all data within a 5-km buffer around each study area so that these observations can serve as outside constraints on the simulations. Residual data are used rather than raw radar measurements to ensure the simulated topography retains long-wavelength BedMachine features. A normal score transformation is then performed on the residuals so that the data conform to standard Gaussian assumptions required by the simulation algorithm. An empirical variogram $\hat{\gamma }(h)$ is produced for each site to quantify spatial covariance or topographic roughness (Fig. 1, C and D). The variogram relates the variance of each pair of residual bed measurements to their separation (lag) distance. The variance increases with lag distance. For example, two bed measurements that are close together typically have a low variance because nearby points often have similar values. However, at large lag distances, the variance is much greater because bed measurements that are far apart are not strongly correlated. The empirical variogram is calculated as

$$\hat{\text{Γ}}(h)=\frac{1}{2N(h)}\;\sum_{\alpha =1}^{N}{\left[b(x_{\alpha })-b(x_{\alpha }+h)\right]}^{2}$$ (16)

where *b*(*x_α_*) is measured bed topography, *x*_α_ is a spatial location, and *N* is the number of point pairs for a given lag distance, *h* (m). Each empirical variogram was calculated with different azimuthal directions to capture any roughness anisotropy. A variogram model is manually fitted to the empirical variogram. For S5 the modeled variogram is

$$\{\begin{array}{c} \text{Γ}(h)=0.4+0.6*\exp(h,1600),h>0\\ \text{Γ}(h)=0,h=0 \end{array}$$ (17)

where exp (*h*, *c*) is the exponential variogram function with a range *c* (m) (*27*). The S5 model variogram has a nugget of 0.4 that represents the short-range variability. No significant topographic anisotropy was found. The RESPONDER model variogram is fitted as

$$\{\begin{array}{c} \text{Γ}(h)=0.1+0.9*\mathrm{sph}(h,2100),h>0\\ \text{Γ}(h)=0,h=0 \end{array}$$ (18)

where sph(*h*, *a*) is the spherical variogram function (*62*). The RESPONDER model variogram has a smaller nugget and larger range than the S5 model variogram, indicating smoother residual roughness at RESPONDER. The RESPONDER model variogram is also isotropic.

The modeled variograms are then used to perform a sequential Gaussian simulation that produces random realizations of phenomena such that the output realization has the same spatial covariance as the input data [e.g., (*27*)]. The sequential Gaussian simulation uses a random path to visit each grid cell and simulate a value. At each grid cell, the variogram is used to estimate the mean and variance of bed, which defines a Gaussian probability distribution. While kriging interpolation will select the mean of the distribution, sequential Gaussian simulation randomly draws from the distribution to generate a simulated value. To ensure a seamless interpolation, each simulated value is constrained by previously simulated grid cells. This process is repeated until every grid cell is populated. This technique has previously been used to simulate the basal topography of Sermeq Kujalleq (also known as Jakobshavn Isbræ) in west Greenland (*30*).

The simulated residual roughness is then back-transformed to recover the original data distribution and added to BedMachine data to produce output DEMs. A Gaussian filter with an SD of 1.5 is applied to remove very short wavelength (≲50 m) topographic features that can cause unrealistic model behavior. The simulation was implemented using the GeostatsPy software package (*63*). For a detailed description of the methodology, see (*27*, *28*) and workflow scripts in the Supplementary Materials. Last, a tapering algorithm detailed in (*23*) is applied to the final 1.6 km of the DEMs to ensure that periodic boundaries have equal elevations and minimize topographic modification. The Supplementary Text outlines why the fitted variogram cannot have a nonzero intercept.

## Supplementary Materials

### This PDF file includes:

Tables S1 and S2 Figs. S1 to S9 Supplementary Text References
