# Supplementary material for: Complex motion of Greenland Ice Sheet outlet glaciers with basal temperate ice
Source: Sci Adv. 2023 Feb 10;9(6):eabq5180. doi: 10.1126/sciadv.abq5180 (PMC9916990; doi:10.1126/sciadv.abq5180)
Supplement: Supplementary file 1 — Tables S1 and S2 Figs. S1 to S9 Supplementary Text References [file sciadv.abq5180_sm.pdf]

Science Advances
AAAS

Supplementary Materials for

Complex motion of Greenland Ice Sheet outlet glaciers with basal temperate ice

Robert Law *et al.*

Corresponding author: Robert Law, robert.law@uib.no

Sci. Adv. **9**, eabq5180 (2023)
DOI: 10.1126/sciadv.abq5180

This PDF file includes:

Tables S1 and S2
Figs. S1 to S9
Supplementary Text
References

Supplementary material for *Complex motion of Greenland Ice Sheet outlet glaciers with basal temperate ice*

Table S1. Details for model runs. * Domain center (longitude, latitude in decimal degrees). † Seed used to simulate topography from model variogram, i = 10007, ii = 10009. ‡ From the average of two boreholes drilled at S5 detailed in (*28*). § northeast quarter of RESPONDER domain.

Run (abbreviation)	Location*	Depth (m)	Slope (°)	*F*	Seed †	*O*
S5 BedMachine (S5bm)	49.2889 W, 67.2014 N (S5 in (*24*))	818‡	0.8	1.2	NA	0.1036
S5 geostat a (S5ga)	-	-	1.05	1.2	i	0.1360
S5 geostat b (S5gb)	-	-	0.9	0.9	i	0.0875
RESPONDER BedMachine (Rbm)	-50.0875 W, 70.5683 N (BH19c in (*58*))	1,043	1.4	1.2	NA	0.1814
RESPONDER geostat a (Rga)	-	-	1.85	1.2	i	0.2396
RESPONDER geostat b (Rgb)	-	-	1.775	0.9	i	0.1724
RESPONDER geostat c (Rgc)	-	-	1.85	1.2	ii	0.2396
SAFIRE geostat (SAFg)	-49.92 W, 70.52 N (S30 in (*17*))	611	3.0	0.9	i	0.2914
resolution tests	§	1,043	1.775	0.9	i	0.1724

Table S2. Model parameters. * Value for Sermeq Kujalleq then Isunnguata Sermia

Symbol	Units	Variable	Value	Citation
A_1	MPa a^{-1}	Rate factor 1	9.133e12	
A_2	MPa a^{-1}	Rate factor 2	7.477e23	
A_{lim}	MPa a^{-1}	Limiting rate factor		
A_s	m a^{-1} MPa^{-n}	Sliding coefficient	2.13e4	Average of Helanow et al. (23) values
C		Maximum slope value	0.16167	Average of Helanow et al. (23) values
C_a	J kg^{-1} K^{-2}	Enthalpy heat capacity A	7.253	Gilbert et al. (61)
C_b	J kg^{-1} K^{-1}	Enthalpy heat capacity B	146.3	Gilbert et al. (61)
E_a	J kg^{-1}	Enthalpy curve parameter a*	1.6155e5 7.5645e4	
E_b	J kg^{-1}	Enthalpy curve parameter b*	-1.5091e5 - 5.8647e4	
E_c	J kg^{-1}	Enthalpy curve parameter c*	1.2341e5 1.1898e5	
G_b	W m^{-2}	Geothermal heat flux	55e-3	Cook et al. (14)
L	J kg^{-1}	Latent heat of fusion of ice	3.34e4	
κ_c	kg m^{-1} a^{-1}	Cold ice enthalpy diffusivity	1.024e-3	Gilbert et al. (61)
κ_t	kg m^{-1} a^{-1}	Temperate ice enthalpy diffusivity	1.045e-4	Gilbert et al. (61)

P_{tr}	MPa	Triple-point pressure of water	0.612	
Q_1	J mol^{-1}	Activation energy 1	60e3	
Q_2	J mol^{-1}	Activation energy 2	115e3	
ρ_i	kg m^{-3}	Ice density	910	
T_{lim}	K	Limit temperature	263.2	Cuffey and Paterson (29)
T_{ref}	K	Reference temperature	200	
T_{tr}	K	Triple-point temperature of water	273.2	
W_1	MPa a^{-1}	Water viscosity factor 1	1.0	
W_2	MPa a^{-1}	Water viscosity factor 2	2.35	
W_3	MPa a^{-1}	Water viscosity factor 3	77.945	
ω		Upper water limit	0.025	

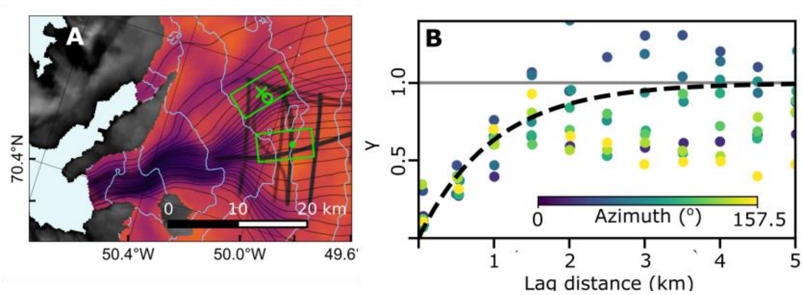

Fig. S1. (**A**) Sermeq Kujalleq showing flowlines in black converging into fjord (pale blue). BedMachine v3 (*12*) basal topography (inferno), land topography (grayscale), and ice surface contours (pale blue). Model domain locations (fluorescent green rectangles) containing RESPONDER BH19c location [fluorescent green cross (*19*)], RESPONDER BH18c location [fluorescent circle (*25*)], SAFIRE BH14b and BH14c locations [fluorescent green dot (*26*)] and radar flight lines for SAFIRE domain [black strokes within dashed boundary, scatter opacity means darker lines have more measurements, (*27*)]. (**B**) modeled variogram (dashed line) and empirical variograms for varying azimuths (scatter points) for SAFIRE domain.

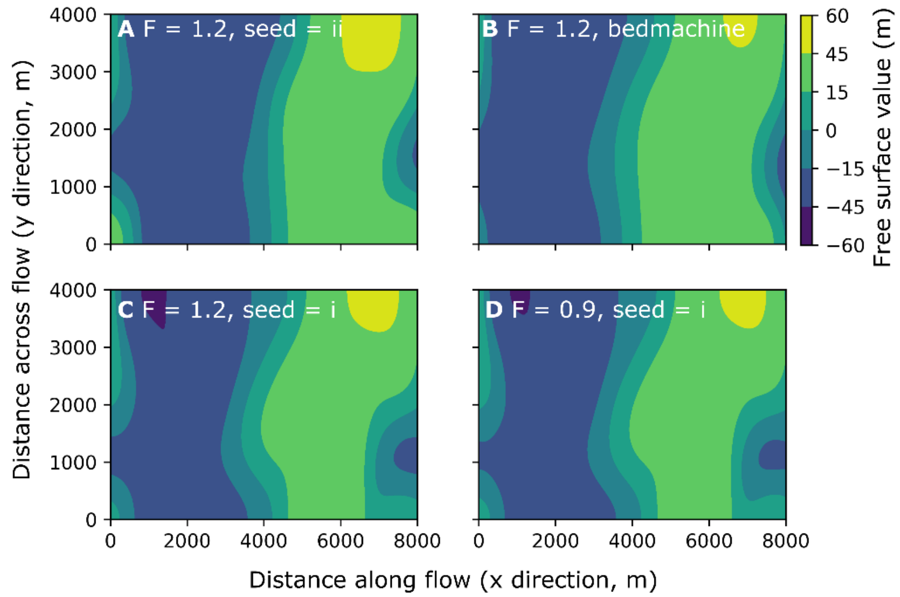

Fig. S2. Free surface variation for RESPONDER runs (**A**) Rgc, (**B**) Rbm, (**C**) Rga, and (**D**) Rgb.

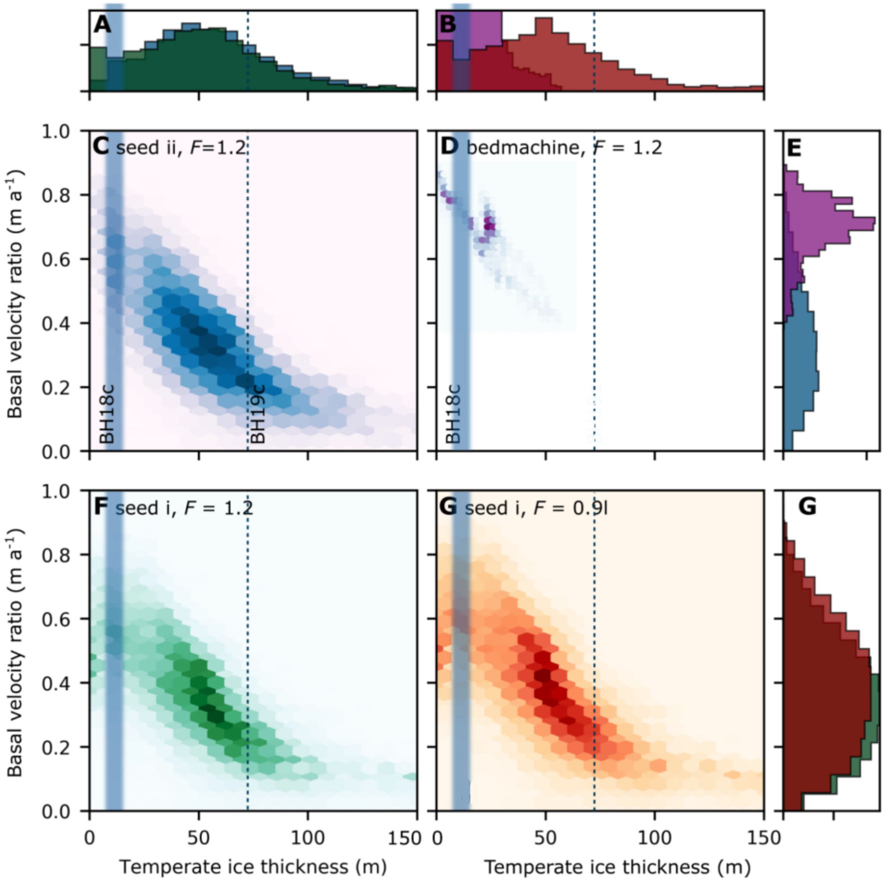

Fig. S3. Hexbins and histograms for four RESPONDER domain runs. Panel details refer to run details. (**C**) = Rgc, (**D**) = Rbm, (**F**) = Rga, (**G**) = Rgb.

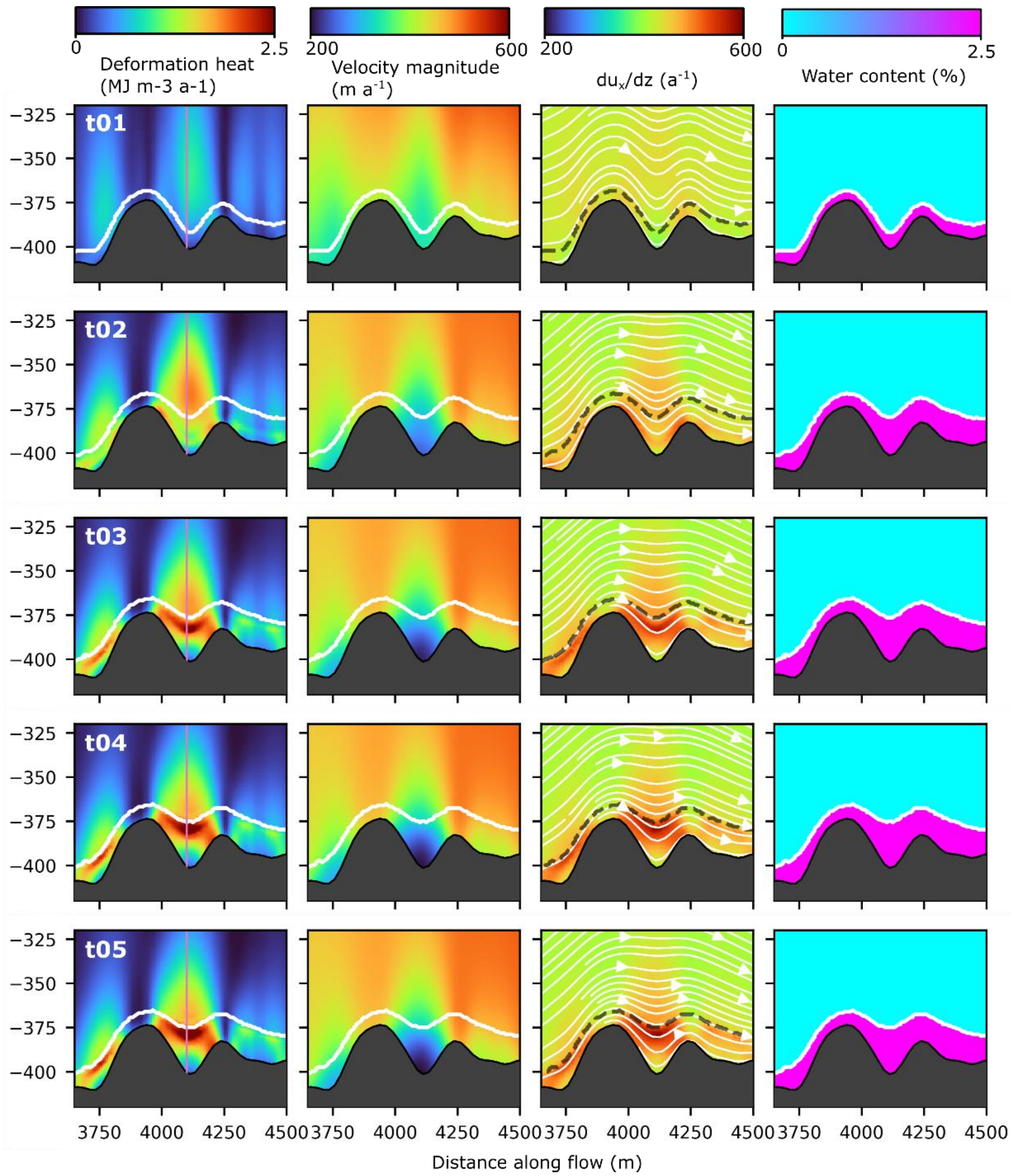

Fig. S4. Evolution towards steady state for deformation heat (column 1), velocity magnitude (column 2), change in x velocity component with height (column 3), and water content (column 4) for the five iterations (rows) required for steady state for the close-up in Fig. 5.

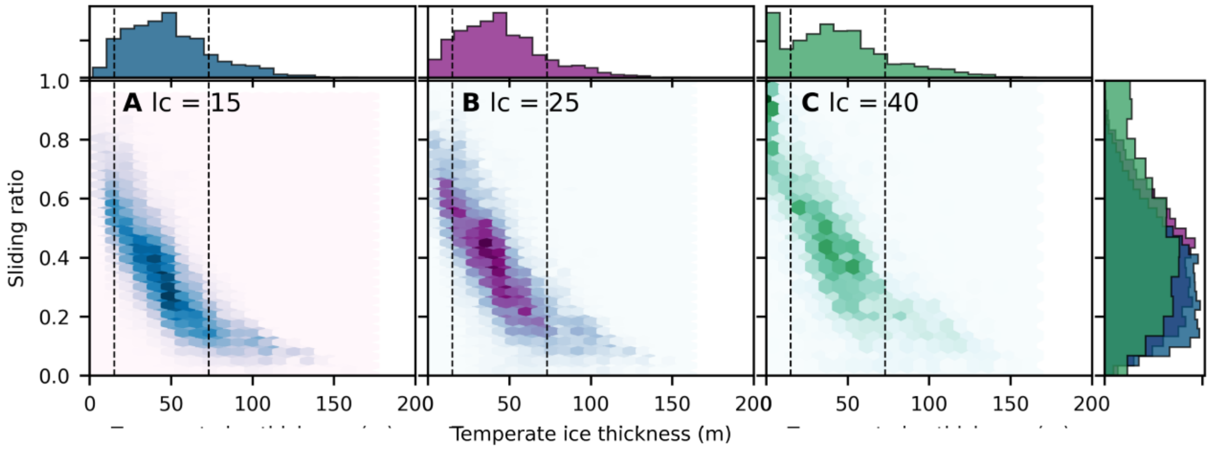

Fig. S5. Hexbins and histograms for representative element length (lc) of 15, 25, and 40 m (A, B, and C respectively). These show minor difference between lc = 15 m and lc = 25 m, with notable increases in temperate ice in the lowest-thickness bin, and high basal velocity ratios for lc = 40 m.

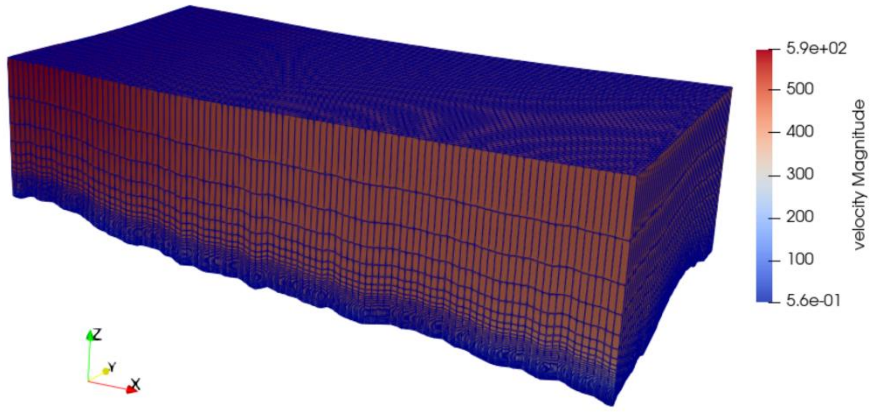

Fig. S6. Domain used in resolution tests, here with lc = 40. Dimensions are 2 km across flow, 4 km along flow, depth ~1,043 m. This is the bottom left corner of the full RESPONDER seed i domain as viewed from above.

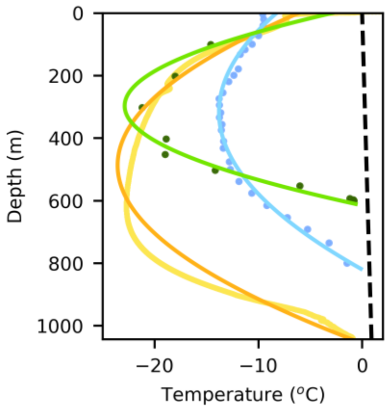

Fig S7. Temperature profiles with fitted curves for RESPONDER (orange), SAFIRE (green), and S5 (sky blue). Yellow line is from BH19c (*19*), green scatter is from (*26*), and blue scatter is from S5 (*28*). Black dashed line is the pressure melting point using a Clausius-Clapeyron slope of 0.0974 K MPa^{-1}.

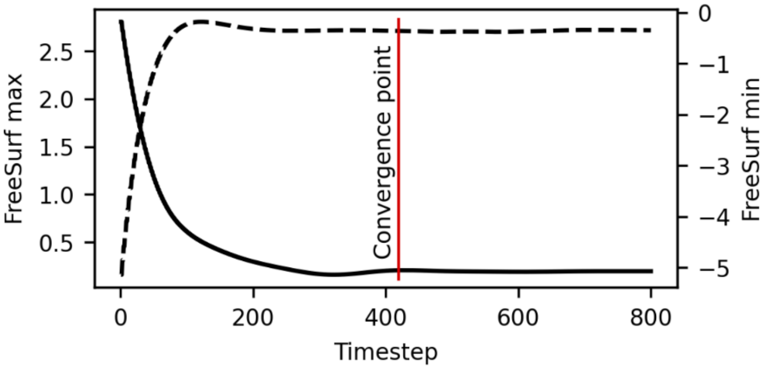

Fig S8. Change in free surface max (dashed line) and min (solid line) values with time including point where the model is deemed to have converged.

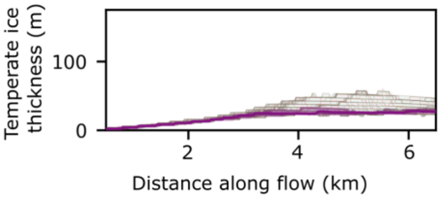

Fig S9. Basal temperate layer ice thickness averaged across flow for the RESPONDER domain forced with BedMachine topography (Run Rbm).

Supplementary text, geological considerations

Individual GrIS glacier catchments are often assumed to be underlain by either hard crystalline bedrock or soft sediments. However, this assumption rests on a paucity of data constraining the physical characteristics of the ice-bed interface. Collection of new GrIS field data over the last decade suggests a more complicated situation, with no clear consensus for blanket hard- or soft-bed settings. At Isunnguata Sermia, borehole data mostly from topographic highs (sites S1-S5) suggests hard-bed conditions (*53*), while seismic surveys from topographic depressions indicate deformable sediment [e.g. (*52*)], and ice-marginal studies for the nearby Russell Glacier indicate basal ice with a high debris content (*66*). At Sermeq Kujalleq, distributed acoustic sensing data from BH19c and seismic surveys across the SAFIRE domain both suggest a layer of sediment (*67*, *68*), yet ice-marginal glacial geology is dominated by areal scour with limited and isolated zones of sediment deposition (*69*). Furthermore, the crystalline Precambrian rock over which both our domains probably lie (*70*) typically exhibits a 'cnoc-and-lochan' landscape in deglaciated areas – characterized by overdeepened glacier-eroded rock basins and knolls with amplitudes up to 100 m (*71*) – as broadly recreated in our geostatistical simulations (Fig. 1).

Our understanding of subglacial transport processes remains poorly quantified (*72*), but we suggest that realistically rough topography, and a variable basal stress/velocity field as produced in our model, are likely to result in heterogeneous sediment distribution and hence heterogeneous basal traction characteristics. High slip rates and basal traction over topographic highs are likely to lead to high rates of erosion, meaning these regions are most likely composed of hard bedrock. Conversely, topographic lows may act as sites of sediment accumulation through basal melt-out, lower bed-normal pressure and slower glacier motion (*73*). This has important implications for basal motion. Although topographic prominences occupy a small overall area they are sites of high basal-slip and the traction at these locations will exert a disproportionate control on basal motion. This variation may explain the success of hard-bed Weertman-style basal-slip relationships across the GrIS when viewed at >1 km scales (*47*) even when more recent studies suggest a regularized-Coulomb approach is more applicable (*23*), particularly if the viscous flow

of temperate ice is contributing a considerable proportion of overall motion. Models investigating spatial variation in basal-traction relationships, similar to (*74*) but also incorporating realistic topography, will shed further light on these processes.

Supplementary text, F values

We use the parameter F as a tool to set O which is included in Eq. 6 through effective pressure, N_e. By using only Eq. 6, with F=1 (i.e. effectively neglecting F in the formulation), O would be theoretically limited to $\frac{\tan(\theta)}{C} \leq O \leq 1$. By including an F of less than 1, O can be less than $\frac{\tan(\theta)}{C}$ (and therefore $\tau_{b_{max}} \leq \tau_d$), which produces stable model results if there is sufficient additional resistance to the driving stress from normal forces from the stoss sides of intermediate-scale obstacles. Conversely, as a result of the very low gradient in τ_b at high basal velocities (Fig. 2B) it becomes necessary to increase F above 1 when the basal topography is very smooth (BedMachine runs) to maintain model stability, though note that O remains well below 1. In practice, the model ceases to converge to reasonable values for RESPONDER BedMachine runs if $F \lesssim 1.15$ and for RESPONDER geostatistical runs if $F \lesssim 0.85$ due to the additional support provided from the stoss side of basal obstacles. To ensure we are sufficiently within the convergence space of the system we therefore set $F = 1.2$ for BedMachine runs, and run geostatistical runs with $F = 1.2$ and $F = 0.9$. This ensures that we are therefore close to maximum possible sliding rates given our domain geometries where force balance is met locally (which is similar to the assumptions of the shallow-ice approximation). Fig. 2B illustrates the range of behavior for τ_b with $F = 1.2$ and $s - b = 1{,}043$ m.

Basal freeze on

In order to hold the basal temperature constant at the melting point the following energy consideration must be met

$$\rho \frac{\partial H}{\partial t} = -\frac{\partial q}{\partial z} + \gamma = \kappa_c \frac{\partial^2 H}{\partial z^2} + \gamma = 0 \qquad \text{(S1)}$$

where q is vertically directed energy flux into the ice and γ accounts for other sources of energy supplied to the bed

$$\gamma = F_b + G_b + Q_b + VHD + \rho V_b L \qquad \text{(S2)}$$

where Q_b is water transport, VHD is viscous heat dissipation (*40*), and V_b is the basal freeze on rate of liquid water. Neglecting terms other than $\rho V_b L$ then gives

$$\rho V_b L = \kappa_c \frac{\partial^2 H}{\partial z^2} \qquad \text{(S3)}$$

and, setting the right hand side to 2.7e-4 (the largest value from the borehole profile of (*19*)(*21*)) gives V_b = 2.8e-5 m a^{-1}, negligible in the context of high basal melt rates modeled across the domain.

Supplementary text, zero intercept of variograms

All the variograms used in this study have a non-zero nugget, where the nugget is a quantitative value representing short scale (~25 m) variability in the topography. The nugget ranges from 0.1 for the RESPONDER domain to 0.4 for the SAFIRE domain (with data normalized to have a variance of 1 meaning these values do not have units). A non-zero nugget occurs when measurements with near-zero lags still have somewhat large variances, where the variance is the y axis of the variogram [although the term ‘semivariance’ may be used in other studies (*64*)]. The variance at a lag distance of zero therefore has to be zero, because a topography value should be 100% correlated with itself. This requirement forces a sharp downward trend in the variograms towards zero.
